# Computing Nucleation Rates from Confined Equilibria: The Critical Cluster Equivalence Principle

**DOI:** 10.1021/jacs.6c09002

**Published:** 2026-08-31

**Authors:** Lunna Li, Fabienne Bachtiger, Aaron R. Finney, Erik E. Santiso, Matteo Salvalaglio

**Affiliations:** † Thomas Young Centre and Department of Chemical Engineering, 4919 University College London, London WC1E 7JE, U.K.; ‡ School of Engineering, University of Liverpool, Liverpool L69 3GH, U.K.; § Department of Chemical and Biomolecular Engineering, 6798 North Carolina State University, Raleigh, North Carolina 27695, United States

## Abstract

Nucleation is a fundamental step in the formation of new materials, with nucleation rates governing phase-transition pathways and outcomes that influence material properties across chemistry, physics, and biology. Nevertheless, extracting nucleation rates remains challenging: experiments are limited by the microscopic length scales involved, while molecular simulations are hindered by the rare-event nature of nucleation and the large system sizes required to sample low-supersaturation regimes typical of experiments and industrial processing. Here, we build on the established thermodynamic correspondence between stable clusters in small, closed systems and critical clusters in open systems and develop a multicomponent extension, the Critical Cluster Equivalence Principle, that transforms this correspondence into a practical workflow for calculating nucleation rates. We employ this equivalence to determine solvent-mediated NaCl crystal nucleation driving forces and rates over a wide range of supersaturations (*S*_NaCl_ ∈ [1.5, 4]) and further validate the framework by benchmarking against curvature-corrected homogeneous nucleation rates for argon vapor condensation. This has been achieved using a small number of brute-force, finite-size simulations in which cluster-size statistics and monomer-exchange dynamics in the steady-state map directly onto critical clusters in macroscopic systems. By combining this multicomponent correspondence with the computational approach developed here, the current investigation has obtained nucleation rates in excellent agreement with experiments and enhanced sampling simulations. This approach provides a unified and generalizable tool for predicting nucleation rates with minimal computational effort, enabling routine application across a wide range of material systems.

## Graphical abstract

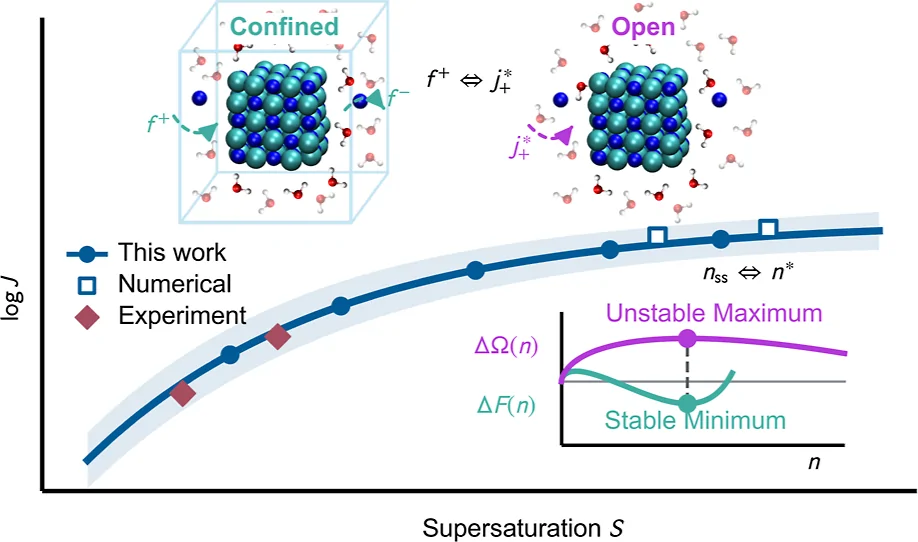

## 1 Introduction

Nucleation underpins the emergence of new phases across physical, chemical, and biological systems. It governs diverse phenomena, ranging from vapor condensation^1–10^ and bubble formation^11–16^ to crystallization^17–27^ and protein self-assembly,^28–33^ thereby shaping microstructure, function, and material properties.

The outcomes of phase separation are governed by the relative nucleation rates of the competing phases. Nevertheless, determining nucleation rates remains a significant challenge.^34,35^ For example, experimental measurements are typically confined to narrow supersaturation windows: at high supersaturation, nucleation proceeds too rapidly to be accurately resolved with standard techniques, whereas at lower driving forces, it becomes very rare and difficult to observe due to the transient nature of critical clusters at the nucleation transition state. This obscures our understanding and ability to control the emergence of out-of-equilibrium phases, particle-size distributions, and, ultimately, the bulk properties of complex systems in which phase transitions play a key role.

Molecular dynamics (MD) simulations offer a powerful means to obtain molecular-level insights into nucleation processes.^36^ However, simulating nucleation in complex systems poses significant challenges. The challenges arise not only from the need to accurately model the multitude of interacting monomers (e.g., molecules or ions) in the surrounding medium but also from the substantial computational costs typically associated with sampling rare nucleation events in brute-force simulations.^37,38^

Recent studies have shown that enhanced sampling methods, including biasing simulations and forward-flux sampling, can significantly accelerate the sampling of rare events,^39–44^ but their application to the formation of complex molecular assemblies or crystals from multicomponent solutions remains limited by the need to identify and efficiently compute variables that faithfully capture nucleation mechanisms.^45,46^ Seeding methods are commonly employed to circumvent the rare-event barrier associated with nucleation; however, as they extract kinetics from finite ensembles of nonequilibrium trajectories around an imposed near-critical nucleus, the resulting rate estimates are inherently noisy and require extensive repeated simulations for convergence, which may be further affected in closed systems by depletion in the driving force.^37,47^ Regardless of the sampling strategy, computing nucleation rates at low supersaturation remains extremely challenging in molecular simulations due to the large system sizes required.

Recent studies of the Ising lattice gas, hard-sphere systems, and water condensation have established a fundamental connection between stable clusters in closed systems and critical clusters in open systems under equivalent driving forces (supersaturation).^48–59^ However, extending from single-component to more complex, multicomponent systems requires a comprehensive thermodynamic foundation.

Herein, we build on the established thermodynamic correspondence between stable clusters in closed thermodynamic systems (such as the canonical ensemble) and critical clusters in open systems (the osmotic ensemble). We extend this correspondence to multicomponent systems—referring to the resulting multicomponent mapping as the Critical Cluster Equivalence Principle (CEP)—and develop a CEP-based thermodynamic–kinetic workflow (Figure 1) to determine nucleation rates for argon condensation and sodium chloride (NaCl) crystallization from aqueous solution.

**Figure 1:**
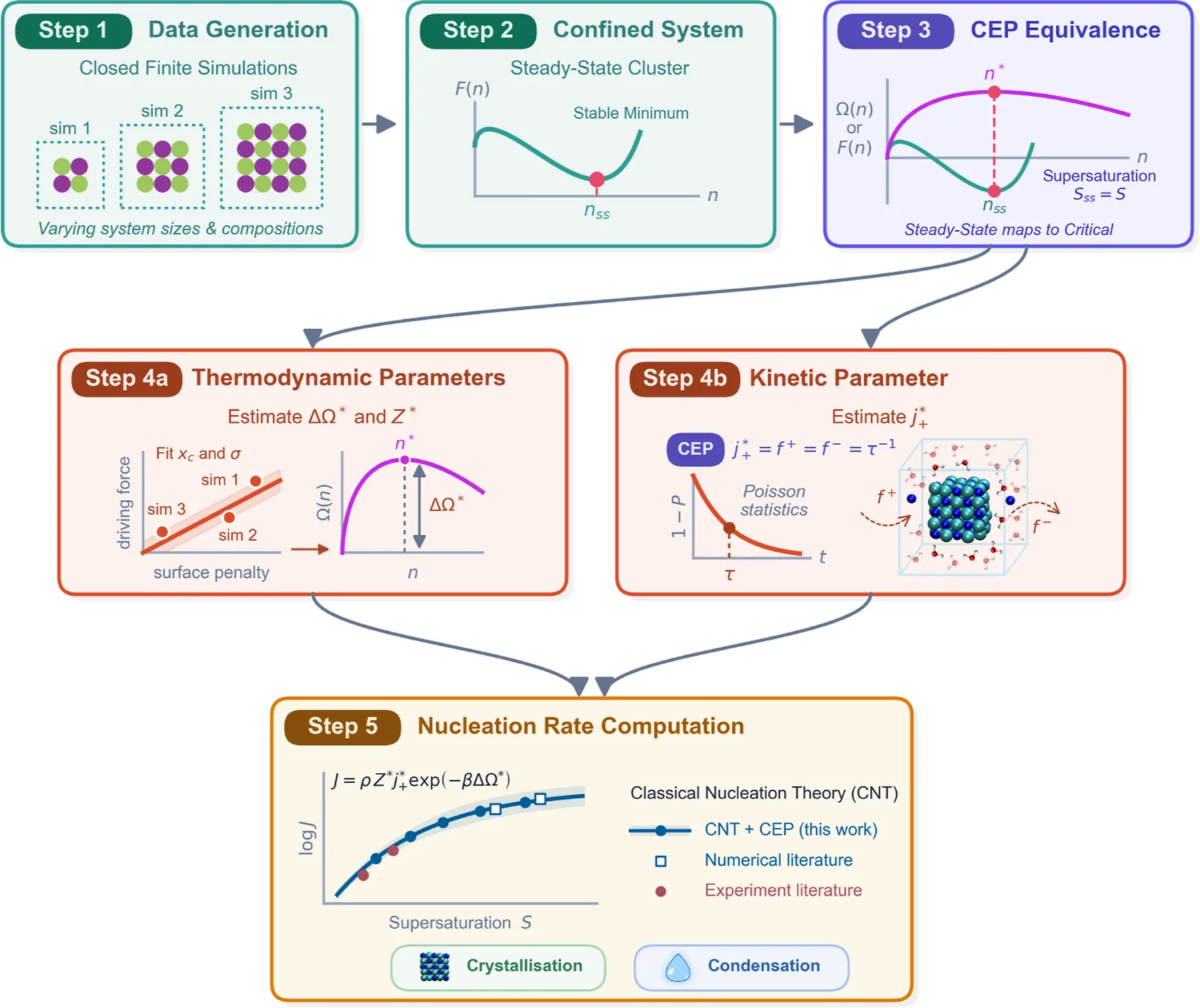
Overview of the Critical Cluster Equivalence Principle (CEP) workflow for computing nucleation rates. Steps 1–2 data generation in confined systems: a small number of independent, closed-ensemble (*NVT*) simulations with varying system sizes and compositions are performed. This allows sampling of stable steady-state clusters, *n*_*ss*_, that reside at the local minimum of the confined free-energy profile, *F*(*n*). Step 3 CEP equivalence: a stable steady-state cluster in a closed system, *n*_*ss*_, maps thermodynamically onto the unstable critical cluster, n*, of an open (osmotic) system experiencing an equivalent supersaturation or chemical driving force. Step 4a thermodynamic parameters: by minimizing the confined residual of the free energy gradient across multiple simulations, fundamental equilibrium parameters, such as coexistence concentration (*x*_*c*_) and surface tension (σ), are extracted. These parameters are used to construct the open free-energy profile, Ω­(*n*), thereby obtaining the nucleation barrier (ΔΩ*) and the Zeldovich factor (Z*). Step 4b Kinetic parameter: the critical attachment frequency, *j*_+_^*^, is estimated from the stationary kinetics of the steady-state cluster. CEP and population detailed balance imply that *j*_+_^*^ equals the steady-state detachment frequency (*f* ^–^), which is evaluated as a rare-event Poisson process using the mean residence time of monomers, τ. Step 5 nucleation rate computation: utilizing the thermodynamic and kinetic parameters derived from the CEP framework, the homogeneous nucleation rate (*J*) is calculated based on classical nucleation theory (CNT). This approach accurately predicts rates across an extensive range of supersaturations for both crystallization (e.g., NaCl from aqueous solution) and condensation (e.g., liquid argon from vapor).

NaCl serves as a prototypical benchmark system to investigate crystallization in multicomponent systems due to its fundamental importance as a binary ionic solid with a well-defined rock-salt structure,^37,47,60–71^ and the availability of experimentally determined nucleation rates.^72–74^ However, a variety of computational methods have been employed to calculate NaCl nucleation rates at distinct supersaturations, without a unified framework that encompasses the entire range of conditions.^37,47,60–71^

By employing CEP, the current work determines critical clusters involved in homogeneous nucleation in open systems from small-scale, brute force simulations at equilibrium in the canonical ensemble. Significantly, attaining steady-state clusters in effectively confined simulations eliminates the need for enhanced sampling techniques to identify the critical states. Furthermore, by leveraging the monomer population detailed balance in the canonical ensemble, attachment and detachment kinetics to and from critical clusters, respectively, can be obtained without explicitly monitoring the evolution of the cluster size during phase separation.

Within the framework of CNT, which describes nucleation as a single-step process, the CEP approach presented in this study enables the determination of homogeneous nucleation rates that are in excellent agreement with experiments and enhanced-sampling simulations. Rates can be obtained over a wide range of supersaturations by using a small number of independent simulations by varying the system size and the number of monomers. This approach allows accurate prediction of nucleation rates at a fraction of the cost of conventional computational methods, providing a generalizable and transferable framework for studying phase transitions in complex systems.

## 2 Results and Discussion

Finite-system thermodynamics and density-functional theory have established a correspondence between stable clusters in closed systems and critical clusters in open systems.^48–59^ Here, we develop a multicomponent extension of this correspondence within a capillary thermodynamic description. In this framework, the reaction coordinate for the nucleation process is the number of monomers *n* of the incipient phase (Table 1). Under a quasi-equilibrium *ansatz*, it is possible to define a relevant thermodynamic potential for every value of *n*. For a multicomponent system in a canonical (*NVT*) ensemble, the relevant thermodynamic potential for a nucleus containing *n* ions is the Helmholtz free energy *F*(*N*_1_, *N*_2_, *V*, *T*, *n*), where 1 and 2 denote ions and water, respectively. This free energy is the sum of contributions from the mother phase *F*_*m*_, the nucleus *F*_*n*_, and the interface *F*_*in*_, such that *F*(*N*_1_, *N*_2_, *V*, *T*, *n*) = *F*_*m*_ + *F*_*n*_ + *F*_*in*_. Using the equimolar dividing surface to define properties of the interface, so that the nucleus contains *n* ions and the mother phase contains (*N*_1_–*n*) ions, the differentials of the three contributions are

$$dF_{m}=-S_{m}dT-pdV_{m}+\mu_{1}d\left(N_{1}-n\right)+\mu_{2}dN_{2}$$

$$dF_{n}=-S_{n}dT-p_{n}dV_{n}+\mu_{n}dn$$

$$dF_{in}=-S_{in}dT+\left[\sigma \left(n\right)\frac{dA}{dn}+A\left(n\right)\frac{d\sigma }{dn}\right]dn$$

where σ is the surface tension and *A* is the surface area of a crystal containing *n* ions. Therefore

1 $$\begin{array}{ll} dF & =-SdT-pdV+\mu_{1}dN_{1}+\mu_{2}dN_{2}+[\left(p-p_{n}\right)\frac{dV_{n}}{dn}\\ & +\left(\mu_{n}-\mu_{1}\right)+\sigma \left(n\right)\frac{dA}{dn}+A\left(n\right)\frac{d\sigma }{dn}]dn \end{array}$$

where the quantity in square brackets is the derivative of the thermodynamic potential with respect to the reaction coordinate *n*. Performing a Legendre transform with respect to *V* and *N*_1_ would yield the driving force of an ensemble at constant *T*, *p*, and the chemical potential of the solute, i.e., the osmotic ensemble.

**Table 1:** Notation Used throughout the Article^a^

symbol	Definition
*n*	size of a cluster (number of solute monomers)
*n*_*ss*_	size of steady-state cluster in a confined system
n*	critical nucleus size in an open system
*r*	radius of a cluster, assuming spherical geometry
*r*_*ss*_	radius of the confined steady-state cluster
r*	radius of the critical nucleus in an open system
*x*	solute molar fraction in solution
*x*_c_	solubility (equilibrium molar fraction at coexistence)
γ	activity coefficient
γ_c_	equilibrium activity coefficient
*S*	supersaturation (general)
*S*_*ss*_	supersaturation experienced by a confined cluster at the steady state
ρ	vapor number density
ρ_c_	equilibrium vapor density at coexistence
v_*n*_	molecular volume of the liquid or crystalline phase
σ	surface tension
σ_Tolman_	Tolman-corrected surface tension
σ_∞_	planar surface tension (Tolman limit)
δ	Tolman length (curvature correction)
ΔΩ(*n*)	osmotic free energy of cluster formation
ΔΩ*	osmotic free energy barrier for nucleation
Δ*G*(*n*)	Gibbs free energy of cluster formation
ΔG*	Gibbs free energy barrier for nucleation
Δ*F*(*n*)	Helmholtz free energy of cluster formation
ΔF*	Helmholtz free energy barrier for nucleation
*f* ^+^, *f* ^–^	attachment and detachment frequencies for a steady-state cluster in a confined system
*j*_+_^*^	attachment frequency for critical nucleus in an open system
τ	mean residence time of a solute monomer in a steady-state cluster
*Z*	Zeldovich factor
*J*	absolute nucleation rate

a Subscript “*ss*” denotes steady-state under confinement; superscript * denotes critical conditions in macroscopic (open) systems.

This holds because, at constant temperature *T* and pressure *p*, fixing the chemical potential of one species in the bulk mother phase unambiguously determines the other remaining via the Gibbs–Duhem relation (*N*_1_dμ_1_ + *N*_2_dμ_2_ = 0). Fixing the solute chemical potential μ_1_ leads to the fundamental relation for the osmotic ensemble

2 $$\begin{array}{ll} d\Omega & =-SdT+Vdp-N_{1}d\mu_{1}+\mu_{2}dN_{2}+[\left(p-p_{n}\right)\frac{dV_{n}}{dn}\\ & +\left(\mu_{n}-\mu_{1}\right)+\sigma \left(n\right)\frac{dA}{dn}+A\left(n\right)\frac{d\sigma }{dn}]dn \end{array}$$

where Ω = *G*–μ_1_ *N*_1_ represents the thermodynamic potential of the osmotic ensemble, i.e., the osmotic free energy. Performing partial differentiation with respect to *n* demonstrates that the stationary points of Ω­(*n*) coincide with those of the Helmholtz free energy *F*(*n*), the thermodynamic potential of the closed (*NVT*) ensemble

3 $${\left(\frac{\partial F}{\partial n}\right)}_{N_{1},N_{2},V,T}={\left(\frac{\partial \Omega }{\partial n}\right)}_{N_{2},\mu_{1},p,T}=0$$

At this point, we have shown that configurations corresponding to a stationary point in a closed *NVT* ensemble must represent equilibrium states in the open, osmotic ensemble. However, their stability is opposite.^49–53^ To discern the causes of this reversal, we further differentiate with respect to *n*.

It is noteworthy that *dp*/*dn* and *d*μ_1_/*dn* vanish in the osmotic ensemble. In a closed system, instead, *dp*/*dn* is generally negligible when the parent phase can function as a barostat for the nascent nucleus. Under isothermal conditions (*dT* = 0), the thermodynamic relation for the nucleus indicates that $\mu_{n}={\mathrm{v̅}}_{n}dp_{n}$ $\left(d\mu_{n}={\mathrm{v̅}}_{n}dp_{n}-sdT\right)$ . The derivative of the nucleus chemical potential with respect to its size thus follows as $d\mu_{n}/dn={\left(\partial \mu_{n}/\partial p_{n}\right)}_{T}\left(dp_{n}/dn\right)={\mathrm{v̅}}_{n}dp_{n}/dn$ . If the nucleus is further assumed to be incompressible, with $V_{n}=n{\mathrm{v̅}}_{n}$ , so that $dV_{n}/dn={\mathrm{v̅}}_{n}$ and *d* ^2^ *V*_*n*_/*dn* ^2^ = 0, subsequently

4a $${\left(\frac{\partial^{2}F}{\partial n^{2}}\right)}_{N_{1},N_{2},V,T}≃-\frac{d\mu_{1}}{dn}+\frac{d^{2}}{dn^{2}}\left[\sigma \left(n\right)A\left(n\right)\right]$$

4b $${\left(\frac{\partial^{2}\Omega }{\partial n^{2}}\right)}_{N_{2},\mu_{1},p,T}=\frac{d^{2}}{dn^{2}}\left[\sigma \left(n\right)A\left(n\right)\right]$$

The surface area scales as *A* ∝ *n* ^2/3^, and we assume an *n*-independent crystalline σ­(*n*) = σ, leading to a negative contribution from the interfacial term. Consequently, the curvature of the osmotic free energy, ∂^2^ Ω/∂*n* ^2^, remains negative regardless of *n*, underscoring the fundamental understanding that the stationary state in an open ensemble corresponds to an inherently unstable critical nucleus.

For liquid nuclei such as argon, deviations from a constant surface tension can be addressed via the Tolman relation,^50,59,75^ σ­(*r*) = σ_∞_/(1 + 2δ/*r*), where δ is the Tolman length. In this case, the second derivative remains negative in the usual regime *r* ≫|δ|.

In contrast, for a closed system, the formation of a nucleus depletes the solute in the mother phase. The derivative of the bulk solute chemical potential with respect to *n* is, therefore, given by

$$\frac{d\mu_{1}}{dn}=\frac{d\mu_{1}}{dx_{1}}\frac{dx_{1}}{dn}=-\frac{x_{2}}{\left(N_{1}+N_{2}-n\right)}\frac{d\mu_{1}}{dx_{1}}$$

where *x*_1_ = (*N*_1_–*n*)/(*N*_1_ + *N*_2_–*n*) and *x*_2_ = 1–*x*_1_ = *N*_2_/(*N*_1_ + *N*_2_–*n*) represent the mole fractions of the solute and solvent, respectively. As *n* → 0, the derivative *d*μ_1_/*dx*_1_ remains finite and positive, while the negative surface term *d* ^2^(σ*A*)/*dn* ^2^ diverges. Conversely, as the nucleus grows with *n* → *N*_1_, the solute mole fraction *x*_1_ → 0. In the dilute limit, the solute chemical potential can be approximated as μ_1_ ∝ ln *x*_1_, so its derivative *d*μ_1_/*dx*_1_ scales as 1/*x*_1_, which results in–*d*μ_1_/*dn* diverging to + ∞. These findings imply that at some intermediate size between *n* = 0 and *n* = *N*_1_, ∂^2^ *F*/∂*n* ^2^ must transition from negative to positive.

To contextualize the CEP in the framework of the CNT, it is convenient to write the free-energy difference between an initial homogeneous phase (*n* = 0) and a configuration containing a nucleus of arbitrary size *n*. In the osmotic ensemble, the exact differential for the free energy with respect to *n* is given by the last term of eq 2.

For an incompressible solid nucleus, the pressure–volume work term $\left(p-p_{n}\right){\mathrm{v̅}}_{n}$ is negligible and can be absorbed into the bulk chemical potential. As a result, the thermodynamic driving force is given by Δμ = μ_*n*_–μ_1_. Integrating *d*Ω from 0 to *n* gives the CNT expression

5 $$\Delta \Omega \left(n\right)\equiv \int_{0}^{n}d\Omega =n\Delta \mu +\sigma \left(n\right)A\left(n\right)$$

For an electrolyte solution such as aqueous NaCl, the chemical potential of a solute ion is given by μ_1_ = μ° + *kT* ln­(*xγ*), where μ° represents the chemical potential of a reference state; *x* ≡ *x*_1_ is the ionic mole fraction, and γ is the mean ionic activity coefficient. At coexistence with the crystal, μ_*n*_ = μ° + *kT* ln­(*x*_c_γ_c_), where the subscript “c” denotes the equilibrium saturation point, giving $\Delta \mu =\mu_{n}-\mu_{1}=-kT\;\ln\left[x\gamma /\left(x_{c}\gamma_{c}\right)\right]$ . Substituting this into eq 5 and applying the geometric constraint for a cubic crystal, where $A=6{\left(n{\mathrm{v̅}}_{n}\right)}^{2/3}$ , $dA/dn=4{\mathrm{v̅}}_{n}^{2/3}n^{-1/3}$ and $d^{2}A/dn^{2}=-\left(4/3\right){\mathrm{v̅}}_{n}^{2/3}n^{-4/3}$ , the complete expression for the free energy of forming an *n*-ion cluster in the open ensemble becomes

6 $$\Delta \Omega =-nkT\;\ln\left(\frac{x\gamma }{x_{c}\gamma_{c}}\right)+6\sigma {\left(n{\mathrm{v̅}}_{n}\right)}^{2/3}$$

Taking the first and second derivatives of ΔΩ­(*n*) with respect to *n* gives

7 $$\frac{d\Delta \Omega }{dn}=-kT\;\ln\left(\frac{x\gamma }{x_{c}\gamma_{c}}\right)+4\sigma {\left({\mathrm{v̅}}_{n}\right)}^{2/3}n^{-1/3}$$

8 $$\frac{d^{2}\Delta \Omega }{dn^{2}}=-\frac{4}{3}\sigma {\left({\mathrm{v̅}}_{n}\right)}^{2/3}n^{-4/3}$$

It is important to note that eqs 7 and 8 are fundamentally identical to the differential relations associated with the open ensemble, i.e., eqs 3 and 4b. Similarly, the Helmholtz free energy Δ*F*(*n*) associated with the formation of a cluster of size *n* is^76^

9 $$\begin{array}{ll} \Delta F\left(n\right) & =-nkT\;\ln\left(\frac{x\gamma }{x_{c}\gamma_{c}}\right)+6\sigma {\left(n{\mathrm{v̅}}_{n}\right)}^{2/3}\\ & +N_{1}kT\;\ln\left(\frac{x\gamma }{x_{0}\gamma_{0}}\right)+N_{2}kT\;\ln\frac{\left(1-x\right)\gamma_{2}}{\left(1-x_{0}\right)\gamma_{2,0}} \end{array}$$

where *x*(*n*) = (*N*_1_–*n*)/(*N*_1_ + *N*_2_–*n*) and *x*_2_(*n*) = 1–*x*(*n*) are the mole fractions of the ion solute and water solvent, respectively, and γ_2_ is the activity coefficient of water. The subscript “0” denotes the state at *n* = 0.

The subsequent two sections will address the CEP for both ideal and nonideal sodium chloride solutions, focusing on the gradient and curvature of Δ*F*(*n*) with respect to *n* in eq 9.

Finally, to relate relevant numbers to experimental standards, it is necessary to depict the driving force in terms of molalities *m* (moles of NaCl formula units per kilogram of water). In experimental studies, the supersaturation of NaCl is typically described as *S*_NaCl_ = *m*/*m*_*c*_. This metric can be correlated with the mole-fraction supersaturation ratio used in the current work, *S* = *x*/*x*_*c*_, by the relation *S*_NaCl_ = *S*(1–*x*_*c*_)/(1–*Sx*_*c*_) (refer to Supporting Information Appendix, Section 1). Because *n* denotes the total number of ions in the crystal, the corresponding number of NaCl units is *n*_NaCl_ = *n*/2.

### 2.1 Equivalence Principle for Ideal Multicomponent Solution

In the ideal-solution approximation, the activity coefficients in the free-energy expressions reduce to unity (γ = γ_2_ = 1). Denoting the confined free energy under the ideal-solution assumption as Δ*F*_id_(*n*), one can determine its stationary points by differentiating eq 9 with regard to *n* ^76^

10 $$\begin{array}{ll} \frac{d\Delta F_{id}\left(n\right)}{dn}= & -kT(\frac{N_{2}}{{\left(N_{1}+N_{2}-n\right)}^{2}}\left[\frac{N_{1}}{x\left(n\right)}+\frac{N_{2}}{x\left(n\right)-1}\right]\\ & +\ln\frac{x\left(n\right)}{x_{c}}+\frac{n\left(-N_{2}\right)}{N_{1}+N_{2}-n}\left(\frac{1}{N_{1}-n}\right))\\ & +4\sigma {\left({\mathrm{v̅}}_{n}\right)}^{2/3}n^{-1/3}\\ = & -kT\;\ln\frac{x\left(n\right)}{x_{c}}+4\sigma {\left({\mathrm{v̅}}_{n}\right)}^{2/3}n^{-1/3} \end{array}$$

where the terms containing *n*, *N*_1_ and *N*_2_ all cancel out.

Upon examination of eqs 10 and 7, an important thermodynamic symmetry can be observed: the free-energy gradient of the closed, finite system conforms to the same functional form as that of the open, macroscopic system, with the only difference being the substitution of the static bulk mole fraction *x* with the instantaneous, depleted mole fraction *x*(*n*), which is coupled to the cluster size *n*. Moreover, imposing the stationary condition *d*Δ*F*_id_(*n*)/*dn* = *d*ΔΩ_id_(*n*)/*dn* = 0 establishes a definitive correspondence at the same cluster size, which is fundamentally aligned with the thermodynamic equivalence between ∂*F*/∂*n* and ∂Ω/∂*n* as presented in eq 3.

To further investigate the equivalence principle, we evaluate the second derivative of the confined free energy

11 $$\begin{array}{ll} \frac{d^{2}\Delta F_{id}\left(n\right)}{dn^{2}} & =kT\frac{N_{2}}{\left(N_{1}-n\right)\left(N_{1}+N_{2}-n\right)}\\ & -\frac{4}{3}\sigma {\left({\mathrm{v̅}}_{n}\right)}^{2/3}n^{-4/3} \end{array}$$

As demonstrated for ∂^2^ *F*/∂*n* ^2^ in eqs 4a, 11 likewise suggests that *d* ^2^Δ*F*_id_/*dn* ^2^ must display a sign change between *n* = 0 and *n* = *N*_1_ (Figure S2). As *d*Δ*F*_id_/*dn* → + ∞ in both *n* → 0 and *n* → *N*_1_ limits, *d*Δ*F*_id_/*dn* = 0 can have no roots, one marginal tangent root, or two roots. When two roots are present, the first is a maximum at a small value of *n*, where the curve is concave down, and the second is a minimum at a larger value of *n* where the curve is concave up. This second extremum does not require any special bias or restraint to simulate—it can be studied in an equilibrium simulation for the closed system. Because the lower- and higher-*n* extrema of the confined free-energy profile have their individual counterparts in open systems corresponding to different concentrations of the solution (eqs 3, 7, and 10), the first extremum is a maximum (therefore unstable) in both ensembles, whereas the second is stable in the closed system but unstable in the open system, indicating that the stable steady-state cluster of the confined system, *n*_*ss*_, directly corresponds to the critical nucleus, n*, of the open system at *n* = *n*_*ss*_ = n* (see Figure S2).

Importantly, this equivalence principle establishes a robust theoretical framework for determining the equilibrium concentration *x*_*c*_ and the liquid–solid surface tension σ of sodium chloride. By sampling the steady-state cluster size *n*_*ss*_ across *M* independent canonical simulations, these fundamental parameters are obtained by minimizing the residual of the first derivative

12 $$\min_{x_{c},\sigma }\sum_{i=1}^{M}{\left[-\ln\frac{x\left(n_{ss,i}\right)}{x_{c}}+4\beta \sigma {\left({\mathrm{v̅}}_{n}\right)}^{2/3}n_{ss,i}^{-1/3}\right]}^{2}$$

where ${\mathrm{v̅}}_{n}$ is the average crystalline volume per ion, and *x*(*n*_*ss*,*i*_) is the ionic mole fraction of the parent solution in the *i*-th canonical ensemble (Table 2).

**Table 2:** Simulations Details for NaCl

*i*	*N*_2,*i*_	*N*_1,*i*_	*n*_*ss*,*i*_	*S*_*ss*,*i*_^id^	*f*_*i*_^–^(ns^–1^)
1	616	276	88	1.89	0.52
2	405	228	114	1.77	0.59
3	1734	658	242	1.56	0.45
4	2158	776	246	1.59	0.44
5	1982	732	254	1.57	0.48
6	1110	528	272	1.51	0.49
7	2775	932	276	1.54	0.49
8	3171	1202	524	1.42	0.52

Equation 12 includes all the approximations of the CEP–capillary derivation: a size-independent surface tension, cubic-crystal geometry, and an incompressible nucleus of constant per-ion volume. The fitted σ is, therefore, an effective capillary parameter rather than a mechanical planar surface tension: because the nucleus and mother phase are partitioned at a sharp dividing surface eq 1, any surface excess, together with any systematic difference between the structurally defined *n*_*ss*_ and the equimolar dividing surface, is absorbed into σ.

Over the NaCl clusters sampled here (Table 2), a constant effective σ gives consistent *x*_*c*_ and σ across the four cluster definitions examined (Figure S3E,F). Predictions extrapolate to smaller critical sizes at higher supersaturation (Figure S8A), where size-dependence and faceted morphology become more important; these are not corrected for in the present study, as no validated size-dependent surface-tension model for the NaCl crystal–solution interface is available.

In each of the *K* bootstrap replicas, an *M*-sized data set is generated by randomly drawing *M* entries, with replacement, from the original {*N*_1,*i*_, *N*_2,*i*_, *n*_*ss*,*i*_} (Table 2), and {*x*_*c*_, σ} are obtained from fitting eq 12. This procedure yields a total of *K* pairs of *x*_*c*_ and σ, with the standard deviations reported as uncertainties. The distribution of the bootstrapped data for M = 8 and *K* = 1000 is shown in Figure 2A,B. The standard deviation of σ becomes stable for *K* ≳ 200 (Figure S3D); we, therefore, employ *K* = 200 for both real-solution models (to be discussed in the next section), with the A1 activity model illustrated in Figure 2C,D. We obtain an equilibrium concentration of *x*_*c*_ = 0.124 ± 0.007 (corresponding to a molality of *m*_*c*_ = 3.9 ± 0.2 mol kg^–1^) and a solid–liquid surface tension of σ = 22.3 ± 3.5 kJ mol^–1^ nm^–2^ (≈37 ± 6 mJ m^–2^). The estimated molality agrees with previous determinations for the JC–SPC/E model: Moučka et al. reported *m*_*c*_ = 3.64 ± 0.20 mol kg^–1^, while Mester and Panagiotopoulos obtained *m*_*c*_ = 3.59 ± 0.04 mol kg^–1^.^62,78^ As these values were obtained from independent chemical-potential and activity-coefficient calculations, the agreement provides additional validation of the CEP-based approach.

**Figure 2:**
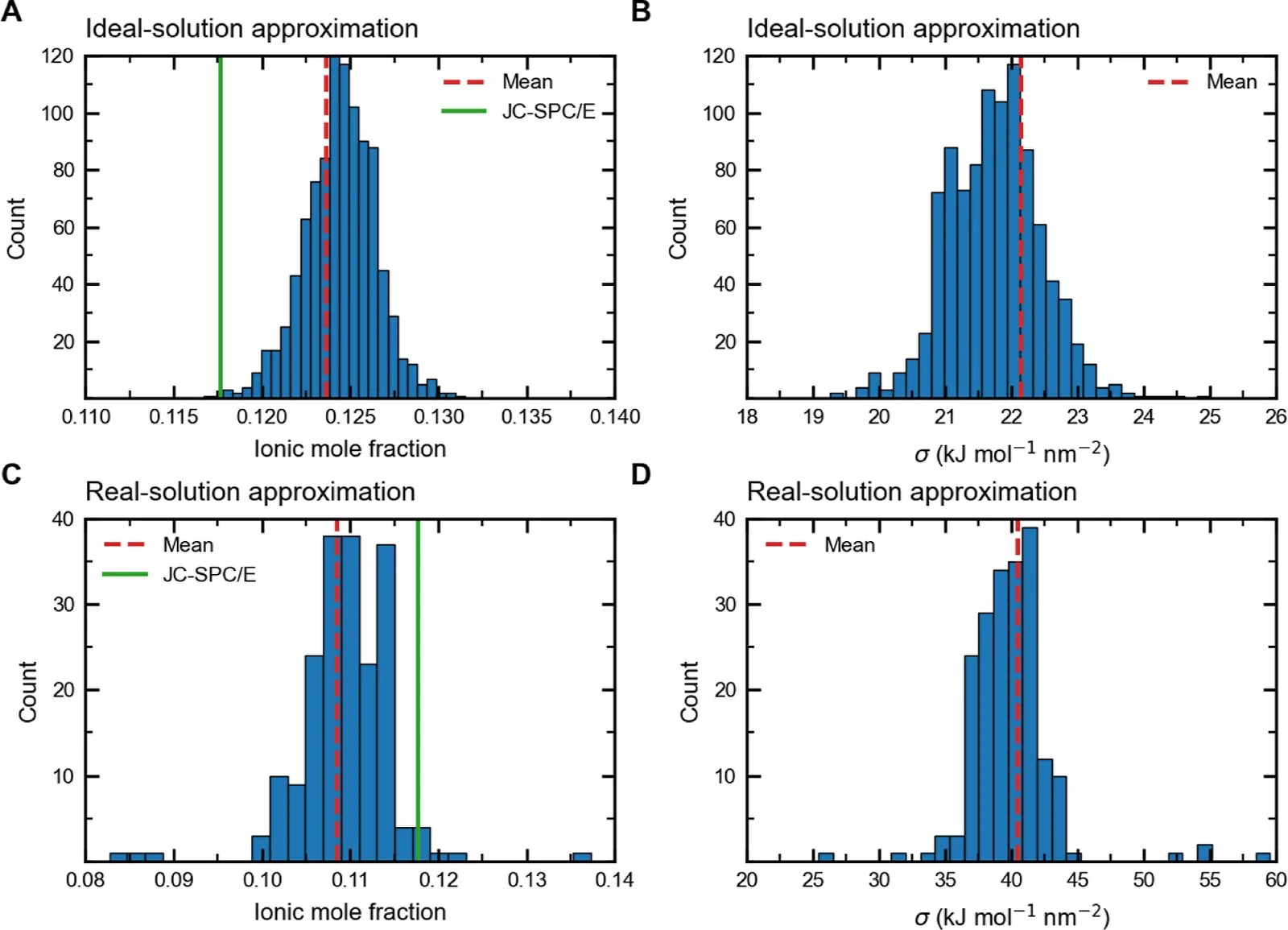
Coexistence concentration and surface tension of JC-SPC/E NaCl: (A,B) for the ideal-solution approximation; (C,D) for the real-solution approximation (A1 activity model). We convert the JC-SPC/E NaCl coexistence concentration obtained from the brute-force simulations *m*_*c*_^ref^ = 3.7 mol kg^–1^ to ionic mole fraction *x*_*c*_^ref^ = 0.1176. The ideal-solution calculation contains 1000 bootstrap samples, and the real-solution calculation contains 200 bootstrap samples.

To identify *n*_*ss*_ for the implementation of eq 12, we employ a comprehensive classification criterion that integrates local ionic coordination numbers (CN) with the per-particle, neighbor-averaged Steinhardt bond-order parameter ${\mathrm{q̅}}_{6}$ (Figures 3A and S1B). To avert spurious misclassification of low-coordinated surface or edge ions, which may display unnaturally high ${\mathrm{q̅}}_{6}$ values due to inadequate neighbor sampling, ${\mathrm{q̅}}_{6}$ is set to zero for ions with a coordination number less than 3. Ultimately, ions that meet the criterion of ${\mathrm{q̅}}_{6}\geq 0.2$ and coordination number (CN) ≥3 are classified as crystalline.

**Figure 3:**
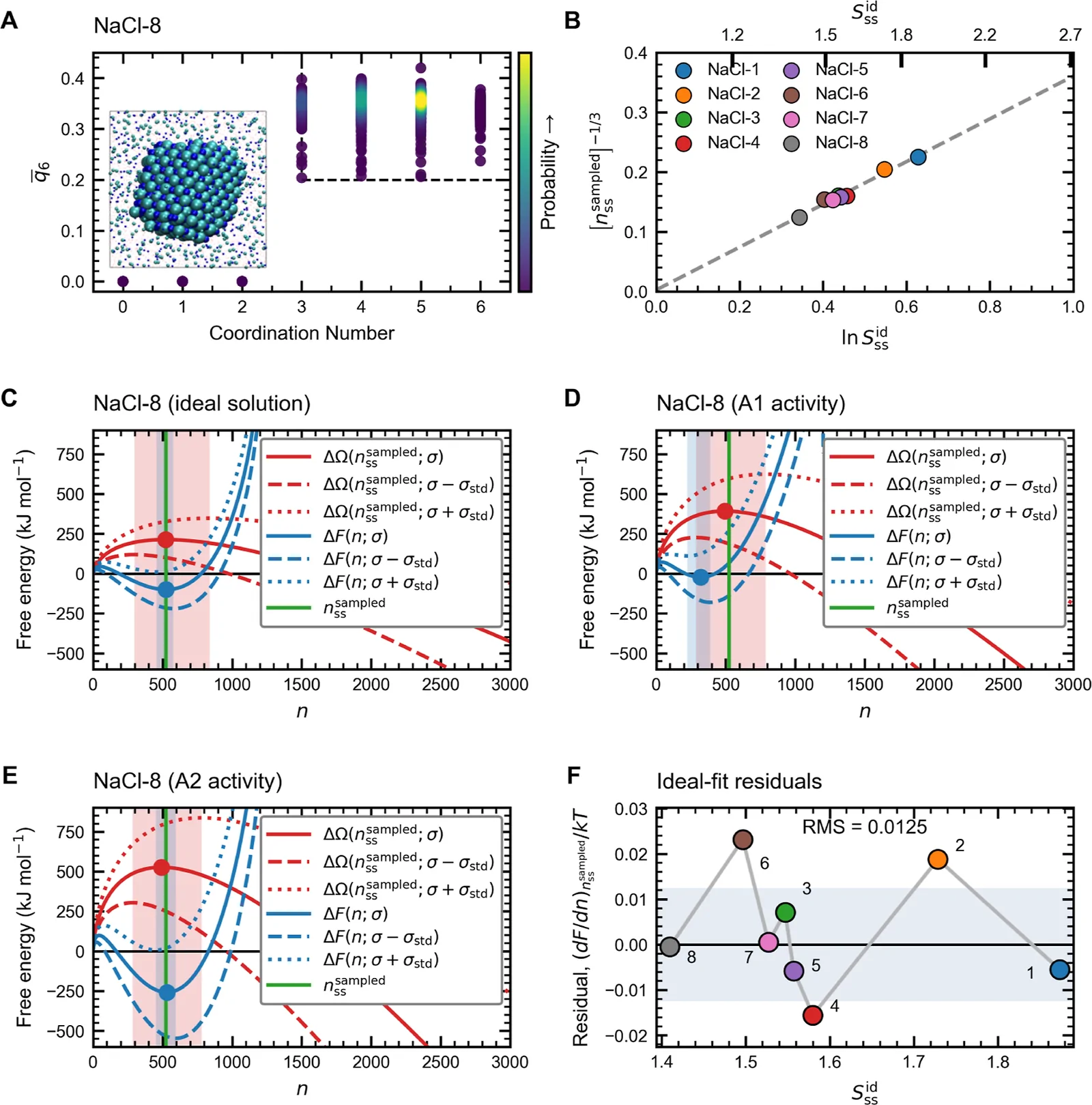
Critical Cluster Equivalence Principle for NaCl at ambient conditions at ideal-solution and real-solution approximations. (A) Two-dimensional classification criterion of ionic coordination number CN and local bond-order parameter ${\mathrm{q̅}}_{6}$ (set to zero for ions with CN < 3), used to identify *n*_*ss*_ for NaCl-8. (B) Linear trend of *n*_*ss*_^–1/3^ versus $\ln S_{ss}^{id}$ for all NaCl systems (Table 2). (C) Reconstructed ideal-solution free-energy landscapes for NaCl-8. Blue curves show the closed finite-bath Helmholtz free energy, Δ*F*(*n*), while red curves show the corresponding open-reservoir osmotic free energy, ΔΩ­(*n*), evaluated using the reservoir concentration associated with the sampled confined cluster size. Solid, dashed, and dotted curves correspond to σ, σ–σ_std_, and σ + σ_std_, respectively. Shaded regions indicate the resulting uncertainty in the fitted positions of the closed-bath minimum and the open-reservoir barrier. The green vertical line marks *n*_*ss*_^sampled^. (D) A1-activity real-solution reconstruction for NaCl-8. The notation and color scheme are the same as in (C). (E) A2-activity real-solution reconstruction for NaCl-8. The notation and color scheme are the same as in (C). (F) Residual quality of the raw eight-data fit for the ideal solution. The stationarity residual ${\left(dF/dn\right)}_{n_{ss}^{sampled}}$ is evaluated at the sampled confined-cluster size for each simulation. A zero residual would indicate that the sampled cluster size coincides exactly with the fitted closed-bath stationary point. The shaded band reports the root-mean-square residual, RMS $=\sqrt{\left\langle r_{i}^{2}\right\rangle }$ , where $r_{i}={\left(dF/dn\right)}_{n_{ss,i}^{sampled}}$ .

We further examine the sensitivity of the results to the chosen cluster definition and order parameter used to identify *n*_*ss*_. In addition to the primary ${\mathrm{q̅}}_{6}/$ CN criterion used in Figure 3A, we conduct cluster analysis using three different definitions: the ${\mathrm{q̅}}_{4t}$ criterion similar to Lamas et al.,^64^ a two-dimensional continuous-CN/*r*_COM_ (center-of-mass distance) free-energy criterion, and a soft hydration-shell/continuous-CN criterion (Figure S1). Two of the three alternative definitions give statistically equal *n*_*ss*,*i*_: the continuous-CN–*r*_COM_ and hydration-shell-continuous-CN definitions lie close to the primary CN– ${\mathrm{q̅}}_{6}$ values, whereas the Lamas-style ${\mathrm{q̅}}_{4t}$ criterion gives systematically smaller clusters (Figure S3F). Nevertheless, when eq 12 is refitted with each alternative set of *n*_*ss*,*i*_, the resulting *x*_*c*_ and σ remain close to the primary values within uncertainties (Figure S3E). This indicates that the inferred coexistence concentration and surface tension are tolerant to clustering methods within the range of definitions examined here.

In addition, we assess the robustness of the results to the number of confined simulations by conducting residual minimization on smaller subsets of the eight NaCl systems. At least five independent simulations are required to obtain averaged *x*_*c*_ and σ parameters that are equal, within uncertainties, to those obtained from the full data set (Figure S3A).

Smaller subsets yield greater σ-uncertainty (Figure S3B). Also, the drift of the fitted σ relative to the full-eight value is largest for subsets that exclude the lowest- and second-highest-supersaturation systems (NaCl-8 and NaCl-2, Table 2), thereby identifying them as the systems that most strongly anchor the fit (Figure S3C). Although NaCl-1 represents the most extreme position on the fit axis (Figure 3B), it does not rank highest in anchoring a stable σ (Figure S3C). This phenomenon is likely associated with its proximity to the state where the two roots of *dF*/*dn* = 0 coalesce into a singular degenerate solution (Figure S2A), resulting in a shallow minimum and consequently the noisiest σ-contribution, so drifted subsets tend to include it rather than exclude it. The eight-data fit thus provides a reasonable and stable estimate of {*x*_*c*_, σ}. The σ uncertainty, and hence the rate uncertainty, could be reduced further by running more confined simulations at the large-*n*_ss_ end of the mother-phase supersaturation range.

By reinserting *x*_*c*_ and σ into the thermodynamic equations, we formulate the confined free-energy profile, Δ*F*(*n*), for each of {*N*_1,*i*_, *N*_2,*i*_, *n*_*ss*,*i*_}. The mathematically derived second stationary point aligns with the *n*_*ss*_ sampled from each simulation (Figures 3C and S6A).

Figure 3B shows that ${\left(n_{ss}^{sampled}\right)}^{-1/3}$ , which is inversely proportional to the linear dimension of the cluster, exhibits a linear correlation with $\ln S_{ss}^{id}$ .

This behavior is consistent with the constant-σ capillary approximation $kT\;\ln S≃2\sigma {\mathrm{v̅}}_{n}/R$ over the sampled cluster-size range.

The residual diagnostic in Figure 3F explains the origin of the apparent stationary-point deviation of ΔΩ­(*n*) from Δ*F*(*n*) in Figure 3C for NaCl-8, and in the other systems (Figure S2).

For a single best-fit set {*x*_*c*_, σ} determined from a finite number of simulations, the residual of eq 12 cannot vanish for all sampled data, which leads to a per-system difference between *n*_*ss*,*i*_^sampled^ and *n*_*ss*,*i*_^*F*^. The ΔΩ­(*n*) profiles in Figures 3C and S2 are built from the sampled mother-phase concentration at *n*_*ss*,*i*_^sampled^; constructing them at the exact *n*_*ss*,*i*_^*F*^ recovers the rigorous CEP mapping (Figure S6A).

### 2.2 Equivalence Principle for Nonideal Multicomponent Solution

The surface tension calculated under the ideal-solution assumption (approximately 37 mJ m^–2^) is comparable to the estimates obtained from seeded MD trajectories, as reported by Zimmermann et al., which relate specifically to the nanosized clusters under nucleation conditions. Nevertheless, this value is much lower than the surface tension of the (100) interface, the predominant plane of nanosized clusters in confined simulations, which was recently determined using the mold integration technique (σ = 104 ± 18 mJ m^–2^) by Sánchez-Burgos and Espinosa.^71^ This disparity may be partially attributed to the finite size and morphology of nanosized clusters compared to the planar limit, as well as the choice of order parameters; however, it may also indicate a fundamental limitation of the ideal-solution approximation. In concentrated electrolytes, strong ion–ion interactions cause significant deviations from ideal-solution behavior. Na et al.^72^ empirically established an activity-based σ of 87 mJ m^–2^, in contrast to a much lower value of 57 mJ m^–2^ derived only from concentration. Consequently, to accurately represent the genuine thermodynamic driving forces, it is essential to integrate mean ionic activities into the CEP framework. To isolate the nonideal component, we define the correction term Δ*F*_corr_(*n*) as the nonideal contribution of eq 9

13 $$\begin{array}{ll} \Delta F_{corr}\left(n\right) & =-nkT\;\ln\left(\frac{\gamma }{\gamma_{c}}\right)+N_{1}kT\;\ln\left(\frac{\gamma }{\gamma_{0}}\right)\\ & +N_{2}kT\;\ln\left(\frac{\gamma_{2}}{\gamma_{2,0}}\right) \end{array}$$

Differentiating with respect to *n* gives

14 $$\begin{array}{ll} \frac{d\beta \Delta F_{corr}\left(n\right)}{dn} & =-\left(\ln\gamma -\ln\gamma_{c}\right)+[(N_{1}\\ & -n)\frac{d\;\ln\gamma }{dn}+N_{2}\frac{d\;\ln\gamma_{2}}{dn}] \end{array}$$

Applying the Gibbs–Duhem relation to the finite parent phase cancels the bracketed activity-coefficient derivative in eq 14 (see Supporting Information Appendix, Section 3). The nonideal first-derivative correction, therefore, reduces to

15 $$\frac{d\Delta F_{corr}\left(n\right)}{dn}=-kT\;\ln\left(\frac{\gamma }{\gamma_{c}}\right)$$

For the confined simulation, activity coefficients defined by ionic mole fraction, γ­(*S*_*ss*,*i*_), and ion-pair molality at the same thermodynamic state can be related; because standard NaCl activity-coefficient models are expressed on the molality scale, we convert between molality and ionic mole fraction (as detailed in Supporting Information Appendix, Section 3).

Integrating the nonideal contribution eq 15 into the confined stationary condition yields the real-solution minimization expression for *M* canonical simulations

16 $$\begin{array}{ll} & \min_{x_{c},\sigma }\sum_{i=1}^{M}\{-\ln S_{ss,i}+4\beta \sigma {\mathrm{v̅}}_{n}^{2/3}n_{ss,i}^{-1/3}\\ & {-\left[\ln\gamma \left(S_{ss,i}\right)-\ln\gamma \left(S=1\right)\right]\}}^{2} \end{array}$$

It is now crucial to determine the expressions for γ and γ_2_, as γ will be required for residual minimization eq 16, while both γ and γ_2_ are needed for the nonideal free energy correction eq 13. We employ the Pitzer–Debye–Hückel model to approximate the mean ionic activity coefficient defined on the ion-pair molality scale *m*, ln γ^*m*^(*m*),^78–80^ and we adopt two distinct approaches to evaluate the excess chemical potentials of solute and solvent in *F*(*n*). In the first thermodynamically consistent approach, referred to as the A1 activity model, a single set of Pitzer–Debye–Hückel parameters is utilized. In this case, the Gibbs–Duhem relation links the solute and solvent activities of the homogeneous mother phase through the osmotic coefficient^78–80^ (see Supporting Information Appendix, Section 4).

In the second approach, referred to here as the A2 activity model, we relax the Gibbs–Duhem constraint to enhance empirical consistency. This approach derives two separate sets of Pitzer–Debye–Hückel parameters by independently solving least-squares minimization problems using NIST experimental values of {ln γ^*m*^, *m*} and {ln *a*_2_, *m*}^80^ (see Supporting Information Appendix, Section 4).

The choice of either activity model has a limited impact on the coexistence solubility but considerably influences surface tension.

Utilizing the A1 activity model gives *x*_*c*_ = 0.11 ± 0.01 (*m*_*c*_ = 3.4 ± 0.3 mol kg^–1^) and σ = 67 ± 9 mJ m^–2^. In constructing the real-solution free-energy profiles *F*(*n*) for the simulated systems, it is observed that the thermodynamic shifts of the parent phase, *N*_1_ *kT* ln­(γ/γ_0_) and *N*_2_ *kT* ln­(γ_2_/γ_2,0_) eq 13, demonstrate divergent trends with increasing cluster size *n*, approaching large positive and negative values simultaneously (see Figure S4).

Small uncertainties in the empirical activity coefficients are, therefore, amplified by their partial cancellation, which often hinders *F*(*n*) from exhibiting stationary points. In order to mitigate the impact of fluctuating factors, we limit the nonideal correction to its leading-order approximation as $\Delta F_{corr}\left(n\right)=-nkT\;\ln\left(\gamma /\gamma_{c}\right)$ . Incorporating this correction into the confined free energy gives

17 $$\begin{array}{ll} \Delta F_{A1}\left(n\right) & =-nkT\;\ln\frac{x\gamma }{x_{c}\gamma_{c}}+6\sigma {\left(n{\mathrm{v̅}}_{n}\right)}^{2/3}\\ & +N_{1}kT\;\ln\frac{x}{x_{0}}+N_{2}kT\;\ln\frac{\left(1-x\right)}{\left(1-x_{0}\right)} \end{array}$$

In the A2 activity model, we determine an equilibrium concentration of *x*_*c*_ = 0.11 ± 0.01 (corresponding to *m*_*c*_ = 3.3 ± 0.3 mol kg^–1^) and a surface tension of σ = 90 ± 13 mJ m^–2^, which aligns closely with the values reported by Na et al.^72^ and Cedeno et al.^74^ Although the correlation between σ and supersaturation remains unexamined, the activity-corrected surface tension exhibits improved agreement with the planar limit (104 mJ m^–2^) reported by Sánchez-Burgos and Espinosa,^71^ thereby highlighting the importance of incorporating nonidealities in the characterization of interfacial thermodynamics. Moreover, this second parametrization approach enables the utilization of the untruncated confined free energy

18 $$\begin{array}{ll} \Delta F_{A2}\left(n\right) & =-nkT\;\ln\frac{x\gamma }{x_{c}\gamma_{c}}+6\sigma {\left(n{\mathrm{v̅}}_{n}\right)}^{2/3}\\ & +N_{1}kT\;\ln\frac{x\gamma }{x_{0}\gamma_{0}}+N_{2}kT\;\ln\frac{\left(1-x\right)\gamma_{2}}{\left(1-x_{0}\right)\gamma_{2,0}} \end{array}$$

Each panel in Figure 3C–E illustrates three cluster sizes: the sampled steady-state size *n*_ss_^sampled^ (Table 2); the confined minimum *n*_ss_^*F*^, reconstructed from {*x*_*c*_, σ} via Δ*F*(*n*); and the open-system maximum n* of ΔΩ­(*n*). Using *n*_ss_^*F*^ as a shared reference identifies two independent discrepancies: (i) the stationary-point residual, *n*_ss_^sampled^–*n*_ss_^*F*^, which quantifies how closely the fit reproduces each sampled data (Figure 3F); and (ii) the rigorous mapping, *n* ^*^–*n*_ss_^*F*^, which measures whether the open profile shares the confined minimum. These two exhibit contrasting behaviors across models. The residuals of the ideal and A2 models are minimal (Figures 3F and S7C), so the three sizes nearly coincide; in contrast, the A1 residual is more substantial (Figure S7B), resulting in a greater deviation of *n*_ss_^sampled^ from *n*_ss_^*F*^ and widening the discrepancy, as shown in Figure 3D. Regarding the rigorous mapping, reconstructing Ω­(*n*) at *n*_ss_^*F*^ gives *n* ^*^ = *n*_ss_^*F*^ for the ideal and A1 models (Figure S6A,B), whereas A2 exhibits a systematic deviation (Figure S6C). This inversion has a single cause: A1’s Gibbs–Duhem-constrained framework links the solvent activity to the solute in the mother phase, ensuring mapping but restricting the shared {*x*_*c*_, σ} too much to accommodate every confined system, whereas A2’s constraint relaxation achieves the opposite effect.

In conclusion, our findings suggest that the fitted coexistence concentration exhibits minimal sensitivity to the activity treatment and remains consistent with independent solubility estimates for the JC/SPC-E model, whereas the surface tension is more sensitive and incorporates the different driving force corrections of the two models.

### 2.3 Attachment Frequencies from the Equivalence Principle

Rates for the formation of critical clusters depend on the frequency of monomer attachment. Traditionally, the evaluation of attachment frequencies to critical nuclei *j*_+_^*^ primarily relies on monitoring the time-dependent mean-square fluctuations of cluster size *n* during seeding simulations.^27,47,60,81,82^ In the current research, we present an efficient method: the direct extraction of *j*_+_^*^ from the stationary kinetics of the steady-state clusters in the canonical ensemble simulations.

A key implication of the established mapping between confined steady-state clusters and open critical clusters is that the probabilities of attachment and detachment are expected to be identical in the ensemble average. Consequently, the population detailed balance implies that the critical attachment frequency *j*_+_^*^ equals the steady-state attachment and detachment frequencies *f* ^+^ and *f* ^–^

19 $$j_{+}^{*}=f^{+}=f^{-}=\frac{1}{\tau }$$

where τ denotes the mean residence time of the incoming solute on the steady-state cluster of the confined system. For a simple, single-component nucleation event such as argon condensation, τ denotes the average lifetime of a particle within the liquid droplet (see Supporting Information Appendix, Section 5). For the crystallization of multicomponent systems such as sodium chloride, τ is defined as the time an ion resides on the surface before either incorporating into the crystalline structure or redissolving into the solution. The steady-state detachment is a rare-event Poisson process, in which each detachment occurs independently and is defined by the rate 1/τ. The cumulative distribution function (CDF), represented as *P*(*t*), characterizes the probability of an ion detaching within a specified time interval *t* and is expressed as follows

20 $$P\left(t\right)=1-\exp\left(-\frac{t}{\tau }\right)$$

where 1–*P*(*t*) represents the survival probability.

This application of population detailed balance to extract microscopic exchange kinetics follows a kinetic rationale similar to earlier studies utilizing master equations. For example, Schaaf et al.^83^ employed confined MD simulations of argon clusters to obtain condensation and evaporation coefficients based on the detailed balance of cluster-size populations. Here, a comparable concept is utilized within the CEP framework. At the mapping of *n*_*ss*_ = n*, the forward and reverse fluxes are balanced, giving *f* ^+^ = *f* ^–^. This represents the stationary-size analogue of the detailed-balance condition used in condensation–evaporation analyses, providing a kinetic prefactor consistent with *n*_*ss*_ and n*.

By analyzing the duration of each ion’s residence on the crystalline surface throughout multiple attachment and detachment cycles (Figure 4A illustrates NaCl-8), we derive the empirical CDFs (ECDFs) for the NaCl-1 to NaCl-8 systems^84^ (Figure 4B). Each formula unit comprises two discrete ions, so the ion-pair attachment frequencies are given by *j*_+,NaCl_^*^ = *f*_NaCl_^+^ = *f*_NaCl_^–^ = 1/(2*τ*).

**Figure 4:**
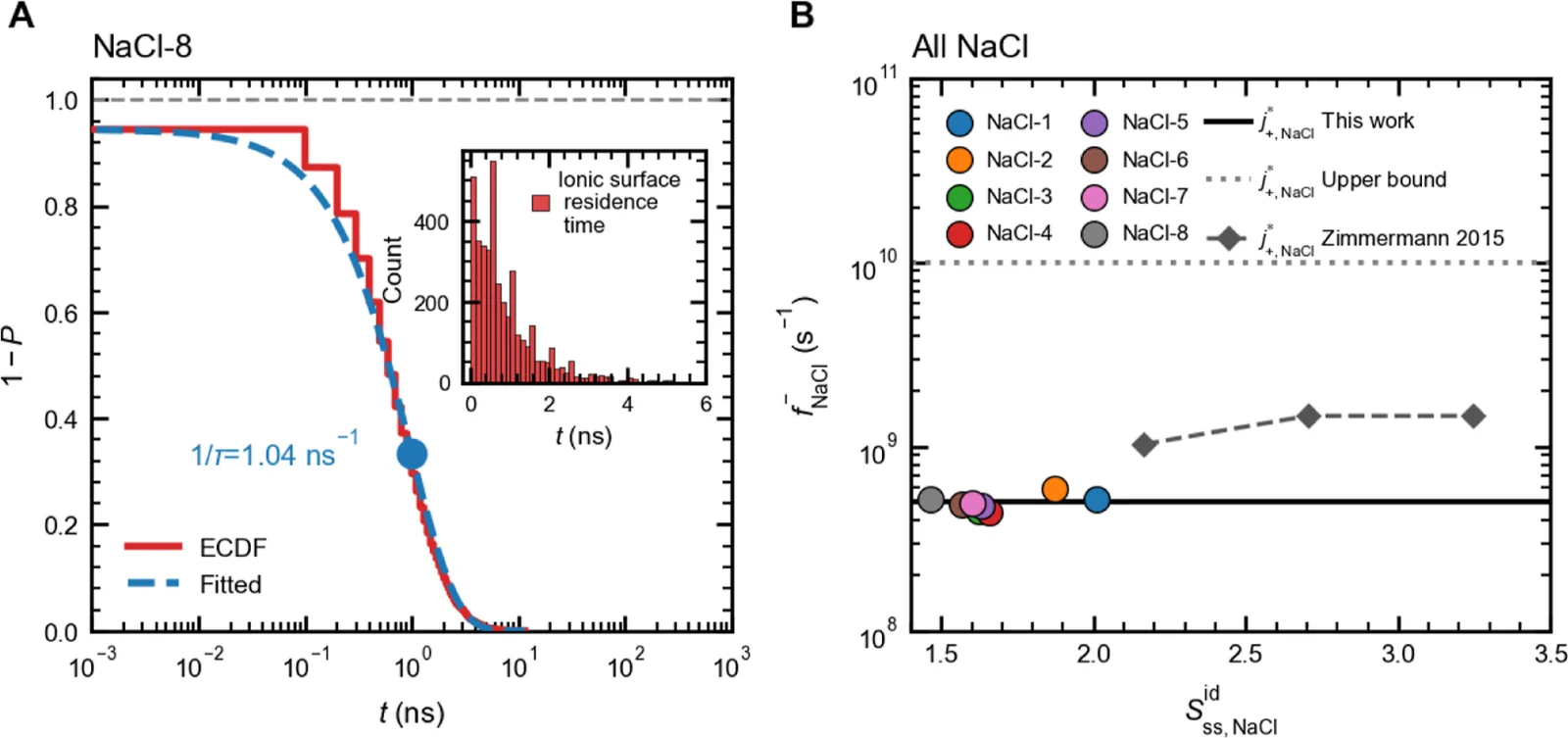
Attachment frequency for critical nuclei. (A) The ECDF of the residence time of ions on the crystalline surface and its Poisson statistics analysis, for NaCl-8. (B) The ion-pair detachment frequencies of the steady-state clusters listed in Table 2, determined using the CEP-based kinetic extraction strategy, along with the upper-bound estimate and literature values of *j*_+,NaCl_^*^.

In excessively small *NVT* systems, finite-size artifacts arising from periodic boundary conditions may perturb attachment and detachment kinetics and interfacial structures. However, our framework inherently mitigates, though it does not eliminate, this risk. Confined thermodynamics dictates the minimum volume of the bulk phase necessary to stabilize a steady-state cluster coupled with its environmental supersaturation *S*_*ss*_.^54,76^ This provides a fundamental baseline for configuring a series of simulation cells in which interfacial kinetics are kept as free from severe periodic self-interactions as is computationally feasible. Nevertheless, this does not completely remove finite-size and sampling limitations: systems with too few parent-phase solutes or too few exchange events may require larger cells or longer trajectories to obtain a converged estimate of τ.

Figure 4B illustrates that the ion-pair detachment rate, *f*_NaCl_^–^, exhibits negligible dependence on the steady-state supersaturation *S*_*ss*_ (under the ideal-solution approximation), maintaining a consistent order of magnitude (≈5 × 10^8^ s^–1^) across all simulated conditions (see also Table 2). In fact, Zimmermann et al.^47^ also reported that as the solution molality changes from 8 mol kg^–1^ to 10 mol kg^–1^ and 12 mol kg^–1^, the corresponding attachment frequencies demonstrate minimal variation, remaining approximately constant at 1.03 ns^–1^, 1.47 ns^–1^, and 1.47 ns^–1^, respectively (Figure 4B).

While the prevalent phenomenon may be associated with a limited supersaturation range in sodium chloride, our findings indicate that the steady-state detachment frequency of argon condensation also shows a minor dependence on the supersaturation. The slight changes are unlikely to significantly affect the calculation of nucleation rates, as the values remain predominantly within a comparable order of magnitude (see Figure S12).

The molecular detachment frequency can alternatively be estimated from the mean first-passage time (MFPT) for surface emission, following Ruckenstein and co-workers,^85,86^ in which emission is modeled as the escape of a surface molecule from the attractive potential of the surrounding cluster. Here, the kinetic prefactor *f* ^–^ derived from the ECDF and Poisson statistics directly samples successful escape events, thereby incorporating both molecular interactions within the droplet and the probability of successful escape at the interface. For argon droplets, the diffusion-based frequency demonstrates that pure diffusion alone does not determine the detachment frequency, because reaching the interface is not equivalent to successful escape (Figures S12 and S13).

Based on a comprehensive review of the existing literature, the upper limit of attachment frequencies can be estimated using the following equation ^[^ ^61,81,87,88^ ^]^

21 $$j_{+}^{*}=\frac{24Dn^{*2/3}}{\lambda^{2}}$$

where λ denotes the mean path length, and *D* denotes the supersaturation-dependent ionic diffusion coefficient, which can be obtained from simulations of NaCl solutions at different molarities in the *NpT* ensemble.^69^ Under standard conditions of supersaturation, *D* is observed to range from 2 × 10^–10^ m^2^ s^–1^ to 7 × 10^–10^ m^2^ s^–1^. For instance, by assuming λ = 10^–9^ m (which approximately corresponds to the scale of molecular dimensions), *D* = 2 × 10^–10^ m^2^ s^–1^, and a small critical nucleus n* = 10, one can approximate an exemplary upper bound of *j*_+,NaCl_^*^ = 1/2*j*_+_^*^≈10 ns^–1^ (Figure 4B).

### 2.4 Nucleation Rates from the Equivalence Principle

#### 2.4.1 Crystallization of NaCl from Solution

According to CNT, the nucleation rate, denoted as *J*, which is defined as the number of critical nuclei formed per unit time per unit volume, is given by

22 $$J_{NaCl}=\rho_{NaCl}Z^{*}j_{+,NaCl}^{*}\;\exp\left(\frac{-\Delta \Omega^{*}}{kT}\right)$$

where ρ_NaCl_ represents the number of ion pairs per unit volume, and $Z^{*}=\sqrt{|\Delta \Omega^{\prime \prime }\left(n\right){|}_{n^{*}}/\left(2\pi kT\right)}$ is the Zeldovich factor. Using the key parameters obtained from the CEP framework, we proceed to calculate the nucleation rate for the crystallization of sodium chloride in accordance with eq 22.

Figure 5A,B shows the predicted nucleation rates for the crystallization of NaCl, employing both ideal-solution (red curve) and real-solution (blue and green curves) approximations across two distinct thermodynamic driving force scales. These predictions are compared with experimental measurements and computational results from various studies. The red curve illustrates the estimates for the ideal solution, whereas the blue curve represents the estimates for the real solution employing a single set of Pitzer–Debye–Hückel parameters (A1 activity model). In contrast, the green curve demonstrates the estimates for the real solution using two sets of Pitzer–Debye–Hückel parameters (A2 activity model). The three curves are presented with their corresponding uncertainties, derived from the standard deviation σ (σ_std_); furthermore, the simulation data from Table 2 are depicted on the curves.

**Figure 5:**
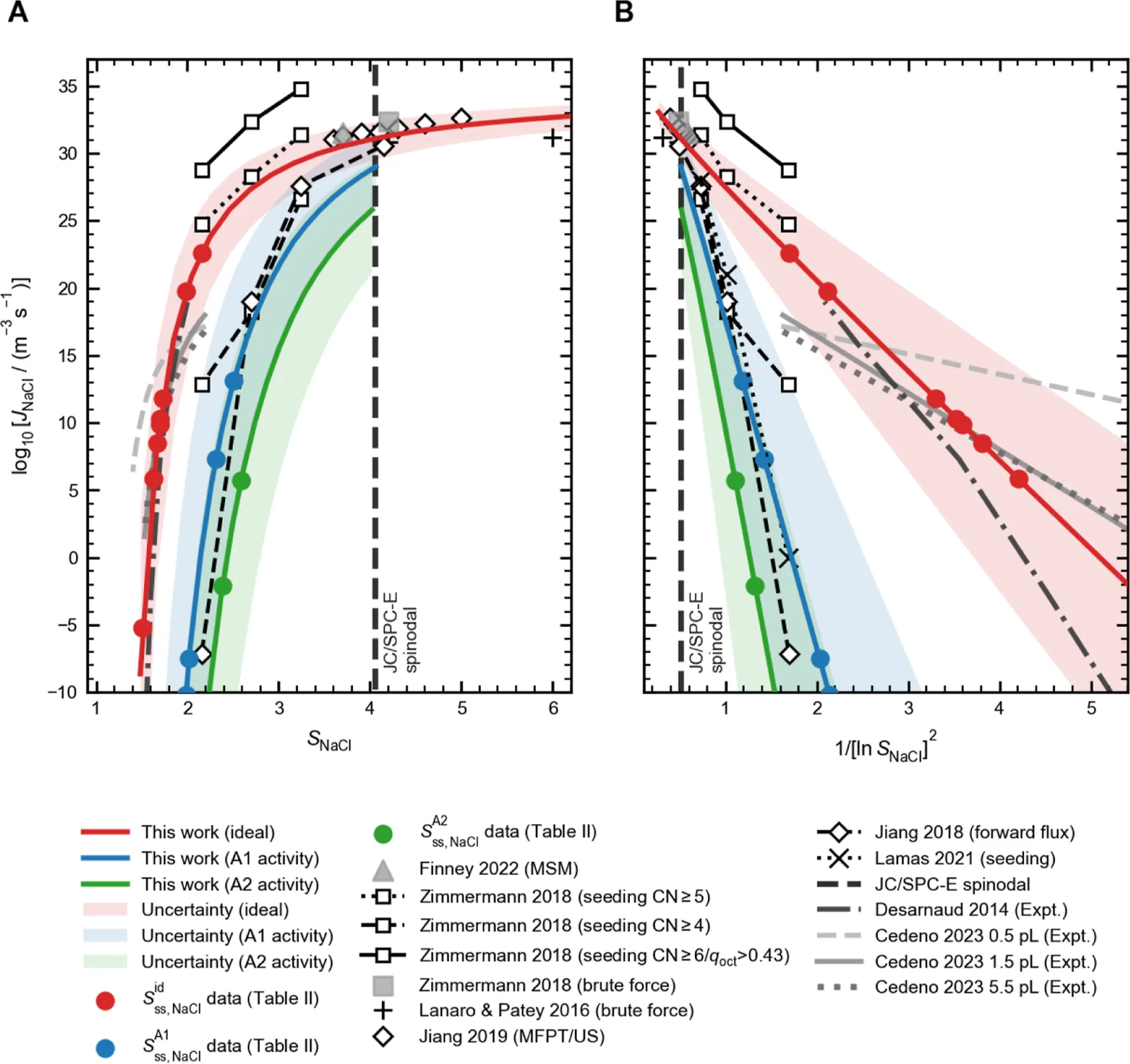
Homogeneous nucleation rates for NaCl crystallization from aqueous solution. (A) Log_10_ of *J*_NaCl_ plotted against the supersaturation *S*_NaCl_. The red curve illustrates the nucleation rates calculated under the ideal-solution approximation. In contrast, the blue and green curves represent the nucleation rates computed for a real solution using different Pitzer–Debye–Hückel parameters. The shaded regions indicate the uncertainty arising from the standard deviation σ_std_ of surface tension σ. The experimental and computational values have been reported in the literature.^37,47,64,66–68,74,77^ All computational values were obtained using the JC/SPC-E force field. The solid circles represent the simulations in Table 2, and the vertical gray dashed line marks the thermodynamic stability limit (spinodal) of the JC/SPC-E NaCl model. (B) Log_10_ of *J*_NaCl_ presented as a function of the reciprocal of the square of the natural logarithm of *S*_NaCl_, 1/ ${\left[\ln S_{NaCl}\right]}^{2}$ .

At elevated supersaturations (*S*_NaCl_ ≥ 3.5), the nucleation rates calculated under the ideal solution assumption align with the existing values in the literature within 2 orders of magnitude. The rates obtained from the A1 activity model remain within roughly 3 orders of magnitude, whereas the A2 activity model gives substantially lower rates. The largest uncertainty associated with the A2 prediction is attributed to its σ standard deviation. Due to the scaling of the CNT-capillary barrier, ΔΩ* ∝ σ^3^/(Δμ)^2^, and its exponential impact on the rate, a one-standard-deviation variation in σ (approximately 15%) propagates into a ∼50% variation in ΔΩ*, and hence a large variation in the predicted rate.

In this regime, the surface free energy penalty gradually decreases with a decrease in critical cluster size (see Figure S5). Conducting experimental measurements in this range of supersaturations presents a challenge, as the solutions typically undergo spontaneous crystallization due to the rapid nucleation rates.

In the intermediate regime (2 ≤ *S*_NaCl_ ≤ 3.5), significant discrepancies are observed among estimates obtained from various numerical methods. An important source of these discrepancies is the identification of crystalline clusters.^37,60^ For instance, Zimmermann et al.^47,60^ demonstrated that reducing the CN threshold from 6 to 4 within the seeding approach can result in a variation of the predicted rates by 10–15 orders of magnitude. Variations in surface tension represent an additional substantial source of discrepancy, alongside solution nonidealities. Importantly, the rates estimated using the A1 activity model demonstrate a strong agreement with the results obtained from the forward flux method.^66^

Under conditions characterized by low thermodynamic driving forces (1.5 ≤ *S*_NaCl_ ≤ 2), rate estimates derived from the ideal-solution model show an exceptional agreement with the empirical data.^74,77^ The rates obtained from activity models are considerably lower than the experimental values. This observation likely indicates the distinction between actual brines and the JC–SPC/E model adopted to simulate NaCl in water. It is essential to note that this low-concentration regime is well-suited for experimental investigations. Nevertheless, unbiased direct nucleation and brute-force simulations, along with certain biased simulations, frequently become excessively slow and computationally demanding. The ability to deliver data within this complex range highlights the significance of the CEP-based workflow.

#### 2.4.2 Argon Condensation with Curvature Correction

After establishing the CEP method for NaCl crystallization, which requires activity-coefficient corrections because of concentrated-electrolyte thermodynamics, we subsequently examine argon condensation as an additional benchmark. This system offers a more straightforward vapor–liquid test case, enabling direct specification of the driving force from the vapor density and allowing evaluation of curvature corrections to the surface tension within the CEP framework. To this end, we employ the same simulation parameters as two prior investigations, each focusing on different supersaturation ranges at *T* = 80.7 K. In Figure 6A, a constant-σ fit reveals an approximately linear correlation between *r*_ss_ and $1/\ln S_{ss}^{id}$ . Nevertheless, a constant-σ value considerably underestimates the nucleation rates at higher supersaturations (blue curve in Figure 6C). Replacing σ with an effective Tolman-like correction,^50,59,75^ σ­(*r*) = σ_∞_/(1 + 2δ/*r*), allows the surface tension to decrease for small critical droplets (Figure 6B), thereby shifting the predicted nucleation rates upward into closer agreement with results acquired independently via metadynamics^89^ and the MFPT method^90^ (Figure 6C). The Tolman-like curvature correction applied here to argon uses a similar finite-bath idea to earlier *NVT*-seeding work, allowing an effective radius-dependent interfacial free energy to be estimated.^59^ We obtain {ρ_*c*_, σ} first and subsequently use ρ_*c*_ to conduct a second fitting procedure for {σ_∞_, δ}. In this second phase, we apply the physical constraint that the surface tension of an argon droplet diminishes with increasing curvature, so that δ > 0. As the bootstrap does not impose this requirement, all samples with δ ≤ 0, together with their associated σ_∞_ are discarded (Figure S9C,D). Notably, while smaller subsets of the complete 11-simulation data set (Table 3) produce large variation in the predicted rate, their median agrees closely with the full-data estimate and the reference values (Figure S9E,F). Additional information regarding the argon systems can be found in Supporting Information Appendix Section 6.

**Figure 6:**
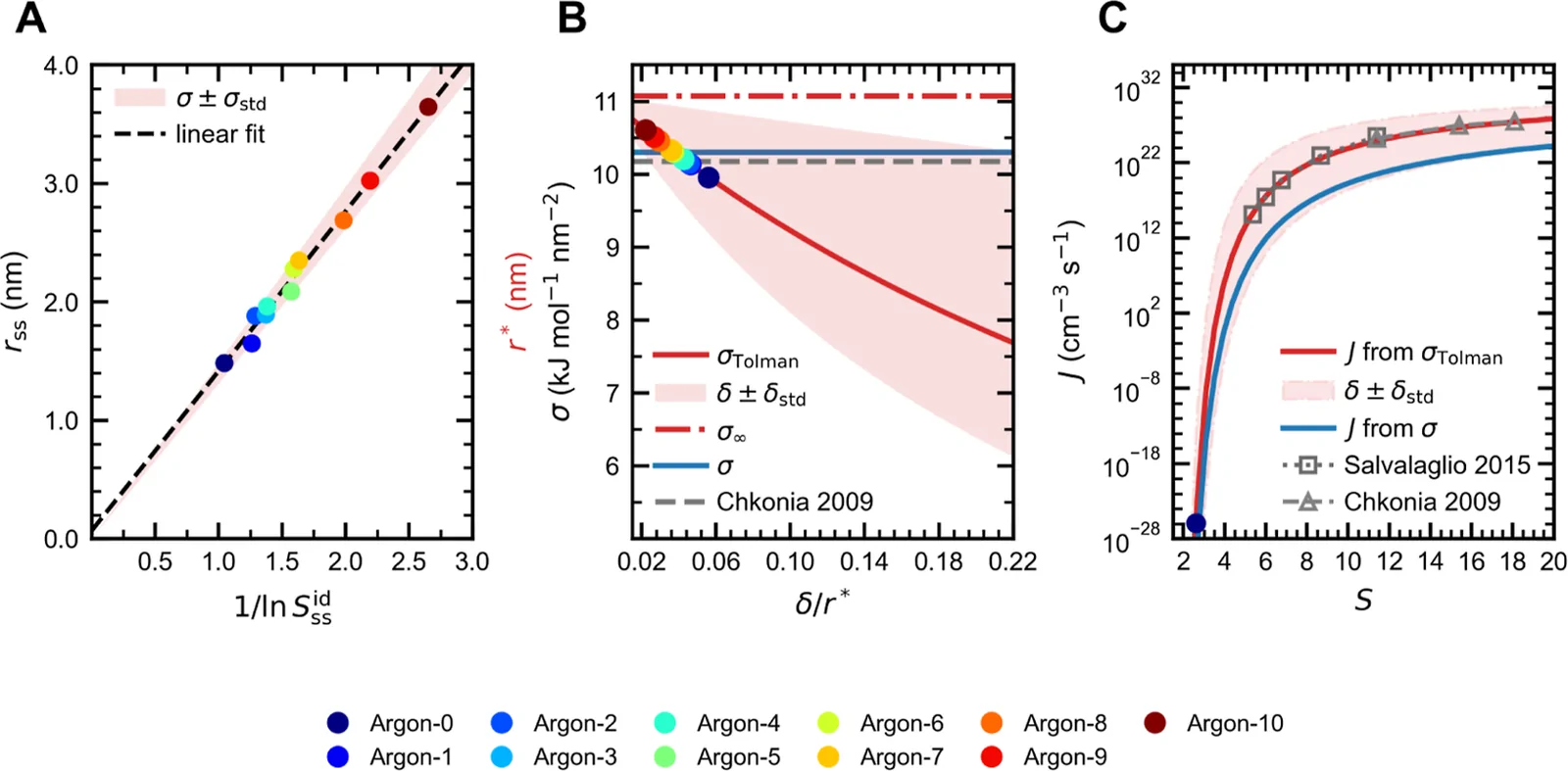
Tolman-corrected confined-equilibrium prediction of liquid-argon nucleation rates. (A) Radius mapping between the confined steady-state droplet radius, *r*_*ss*_, and the inverse supersaturation coordinate, $1/\ln S_{ss}^{id}$ , for the 11 confined argon simulations. The dashed line is a linear fit, and the pink region indicates the uncertainty expected from the standard deviation of the fitted constant surface tension. The right–hand axis gives the corresponding critical radius r*. (B) Tolman-corrected surface tension, σ­(*r*) = σ_∞_/(1 + 2δ/*r*), plotted as a function of δ/r*. The red curve shows the fitted Tolman-corrected σ­(*r*), the pink region indicates the uncertainty propagated from δ, and the horizontal lines show the fitted planar-limit σ_∞_, the constant-σ estimate, and the Chkonia reference value.^90^ (C) Nucleation-rate prediction as a function of supersaturation. The Tolman-corrected prediction is compared with the constant-surface-tension prediction and with literature rate estimates from Salvalaglio et al.^89^ and Chkonia et al..^90^

**Table 3:** Argon Simulations at *T* = 80. 7 K

*i*	*L*_*i*_ (nm)	*N*_*i*_	*n*_ss,*i*_	*r*_*ss*,*i*_ (nm)	*S*_ss,*i*_	*f*_*i*_^–^(ns^–1^)
0	7.5	343	302	1.5	2.6	1.05
1	10.5	512	415	1.7	2.2	0.81
2	7.5	648	615	1.9	2.2	0.65
3	11.0	729	625	1.9	2.1	0.61
4	7.5	729	698	2.0	2.0	0.55
5	13.1	1000	839	2.1	1.9	0.51
6	15.0	1331	1091	2.3	1.9	0.48
7	12.5	1331	1196	2.4	1.8	0.44
8	15.0	2000	1790	2.7	1.8	0.35
9	15.0	2744	2546	3.0	1.6	0.31
10	14.1	4624	4478	3.6	1.4	0.17

## 3 Conclusions

Despite its central importance to the formation of new materials, predicting nucleation rates remains a major challenge due to the difficulty of accessing critical states. While computational approaches for sampling critical clusters are available, they are often prohibitively expensive for determining nucleation rates in multicomponent systems across a wide range of supersaturations, limiting the ability to validate models and predict phase separation outcomes. Here, we expand upon the established thermodynamic correspondence between stable clusters in small, closed systems and critical clusters in open systems, and introduce a multicomponent extension of this relationship, which we refer to as the CEP. Applying the CEP-based workflow to equilibrium states from a small number of small-scale brute-force molecular simulations, we determine the homogeneous nucleation rates for sodium chloride solution and liquid argon over a wide range of supersaturations, in excellent agreement with benchmark results from experiments and simulations. The present CEP workflow is a CNT–capillary construction. For argon, a Tolman-like correction allows the surface tension to vary with cluster size, but this remains a capillary description over a limited droplet-size range and does not guarantee that all finite-curvature, diffuse-interface, and dividing-surface effects are captured. For sodium chloride, we retain a size-independent σ, as no validated size-dependent model is available for the crystal–solution interface; thus the fitted σ is also an effective capillary parameter rather than a purely mechanical planar surface tension, absorbing the surface excess and any discrepancy between the structurally defined *n*_*ss*_ and the equimolar dividing surface. The framework further requires accurate ion and water activity models that can be evaluated, within the free-energy expressions, at the solution conditions of each confined steady-state cluster, as these activities are a substantial source of uncertainty in the predicted rates. In conclusion, this framework provides a general and computationally efficient route to predicting nucleation rates in complex systems, enabling systematic access to phase behavior and emergent material properties.

## 4 Materials and Methods

### 4.1 Simulations of Argon

A total of 11 argon systems were simulated using the Lennard-Jones potential with parameters ϵ = 0.99797 kJ/mol and Σ = 0.3405 nm at *T* = 80.7 K in the canonical *NVT* ensemble^89^

23 $$V_{LJ}\left(r\right)=4\epsilon \left[{\left(\frac{\Sigma }{r}\right)}^{12}-{\left(\frac{\Sigma }{r}\right)}^{6}\right]$$

The potential was truncated but not shifted at a cutoff length of 6.75 Σ. The leapfrog integration time step for the equations of motion was 5 fs, and the temperature was maintained using the Bussi–Donadio–Parrinello thermostat^91^ with a time constant of 0.1 ps. After the systems reached equilibrium, which took approximately 50–100 ns depending on system size, trajectories were sampled every 100 steps for approximately 100 ns. For the largest system, argon-10, the trajectory was sampled every 1000 steps for 150 ns. All simulations were performed in GROMACS 2019.04 ^92^ with PLUMED 2.7.^93,94^

### 4.2 Simulations of NaCl Solution

Consistent with other recent simulation studies of NaCl nucleation,^69,70^ NaCl solutions were modeled using the Joung and Cheatham force field^95^ together with the SPC/E water model.^96^ The geometry of water molecules was constrained using the LINCS algorithm.^97^ A total of eight NaCl systems were simulated in the *NVT* ensemble at *T* = 298 K for between 2 and 7 μs, depending on the system size and the equilibration state of the initial seeded crystal. We pre-equilibrated each system in the *NpT* ensemble at *T* = 298 K and *p* = 1 bar for 1 ns, using a Parrinello–Rahman barostat^98^ and a compressibility of 4.5 × 10^–5^ bar^–1^. The temperature was maintained using the Bussi–Donadio–Parrinello thermostat.^91^ All simulations were performed with GROMACS 2019.04 ^92^ using a 1 fs leapfrog integration time step. Electrostatic interactions were computed using Particle Mesh Ewald summation.^99^ Real-space contributions were considered for atoms within 0.9 nm, while Lennard-Jones interactions were truncated at 0.9 nm. Additionally, dispersion corrections were included to account for short-range intermolecular interactions.

## Supplementary Material



## Notes

Derivation of the relation between mole-fraction supersaturation *S* and molality supersaturation *S*_NaCl_; alternative steady-state cluster definitions for NaCl, including two-dimensional continuous-CN/*r*_COM_ and soft hydration-shell free-energy surfaces, radial distribution functions, and the Lamas-style unnormalized ${\mathrm{q̅}}_{4}$ criterion; Gibbs–Duhem simplification of the real-solution correction and conversion between molality- and mole-fraction-scale activity coefficients; Pitzer–Debye–Hückel activity models with thermodynamically consistent (A1) and decoupled (A2) parameter sets; critical-cluster equivalence mapping for the ideal-solution NaCl systems; sensitivity of the fitted coexistence concentration and surface tension to the number and choice of confined simulations, bootstrap statistics, and cluster definition; real-solution equivalence mapping for the A1 and A2 activity models; stationary-point consistency checks and stationarity residual diagnostics for the ideal, A1, and A2 models; ideal- and real-solution nucleation components as functions of *S*_NaCl_; multicomponent equivalence derivation, modified-liquid-droplet and Tolman-corrected free-energy expressions for argon, with bootstrap and subset diagnostics and Tolman-corrected equivalence mapping; confined detachment-rate and diffusion analysis, including Poisson-based and mean-first-passage-time estimates, coordination-number and mean-squared-displacement data, and per-system argon diffusion parameters (PDF)
