# Supplementary material for: Computing Nucleation Rates from Confined Equilibria: The Critical Cluster Equivalence Principle
Source: J Am Chem Soc. 2026 Aug 31;148(36):38829–44. doi: 10.1021/jacs.6c09002 (PMC13587681; doi:10.1021/jacs.6c09002)
Supplement: Supplementary file 1 [file ja6c09002_si_001.pdf]

Computing Nucleation Rates from Confined Equilibria: The Critical Cluster Equivalence Principle
Supplementary Information

Lunna Li,[1] Fabienne Bachtiger,[1] Aaron R. Finney,[2]
Erik E. Santiso,[3] and Matteo Salvalaglio[1,*]

[1] *Thomas Young Centre and Department of Chemical Engineering, University College London, London WC1E 7JE, United Kingdom*

[2] *School of Engineering, University of Liverpool, Liverpool L69 3GH, United Kingdom*

[3] *Department of Chemical and Biomolecular Engineering, North Carolina State University, Raleigh, North Carolina 27695, United States*

(Dated: August 7, 2026)

S1. RELATION BETWEEN $S = x/x_c$ AND $S_{\mathbf{NaCl}} = m/m_c$

The total ionic mole fraction, as expressed in the main text, is given by,

$$x = \frac{N - n}{N_2 + N - n}$$

And molality is defined as,

$$m = \frac{n_{\text{NaCl}}}{\text{kg of water}} = \frac{n_{\text{NaCl}}}{n_2 M_2}$$

where n_{NaCl} is the moles of NaCl ion pairs, and $n_2 = 1/M_2 = 55.5$ mol kg^{-1} is the number of moles of water per kilogram, with $M_2 = 0.018$ kg mol^{-1}.

The ionic mole fraction, x, can be expressed in terms of n_{NaCl} and n_2,

$$x = \frac{2n_{\text{NaCl}}}{2n_{\text{NaCl}} + n_2}$$

Substituting $\frac{n_{\text{NaCl}}}{n_2} = \frac{x}{2(1-x)}$ gives,

$$m = \frac{1}{M_2}\frac{x}{2(1-x)}$$

At coexistence,

$$m_c = \frac{1}{M_2}\frac{x_c}{2(1-x_c)}$$
$$= \frac{n_2 x}{2(1-x)}$$

The above equations can be used to relate S_{NaCl} and S:

$$S_{\text{NaCl}} = \frac{m}{m_c} = \frac{\frac{1}{M_2}\frac{x}{2(1-x)}}{\frac{1}{M_2}\frac{x_c}{2(1-x_c)}} = \frac{x}{x_c}\frac{1-x_c}{1-x} = S\frac{1-x_c}{1-Sx_c}$$

* m.salvalaglio@ucl.ac.uk

S2. ALTERNATIVE STRATEGY FOR COMPUTING n_{ss}

In Fig. S1A, instead of using integer coordination numbers and the local bond-order parameter $\overline{q}_6$, we also employed an alternative approach to identify crystalline ions for calculating n_{ss}. We carried out a two-dimensional free-energy surface analysis, following the same approach applied to argon. The first collective variable, the continuous coordination number, was defined as $c_i = \sum_{j \neq i} f(r_{ij})$ where r_{ij} denotes the distance between particles i and j and $f(r_{ij})$ is a continuous switching function, computed again using `PLUMED 2.7`. Based on the ion-ion radial distribution function, $r_0 = 5.0$ nm was used with an NLIST cutoff of 10.0 nm. The second collective variable was the distance between individual ions and the COM of the crystal. All the n_{ss} values computed in this manner agree with those obtained using the first method within statistical uncertainties.

In Fig. S1B, from the ion–ion RDF, a cutoff distance of $r_{\mathrm{cut}} = 0.35$ nm was used to construct neighbour lists, evaluate coordination numbers, and compute the per-particle, neighbour-averaged Steinhardt bond-order parameter $\overline{q}_6$ using `Freud 2.8.0` [1].

In Fig. S1C, we computed the Lamas-style unnormalised $\overline{q}_4$ order parameter for each ion in each configuration frame, and treating Na^+ and Cl^- as identical particles and using the same 4 Å neighbour cutoff as in the Lamas definition. The Lechner–Dellago neighbour-averaged $l = 4$ order parameter was used without dividing by the number of neighbours plus one, following the unnormalised definition proposed for NaCl. The resulting $\overline{q}_4$ values were plotted with the coordination numbers from the $\overline{q}_6$/CN analysis; the dashed vertical line marks the Lamas threshold $\overline{q}_{4,\mathrm{t}} = 3.06$. For panel D, we constructed an independent hydration-shell descriptor using the PLUMED-style continuous ion CN and the normalised soft water coordination number, where the soft water oxygen CN of each ion is normalised by its solution hydration value. A two-dimensional histogram was converted into a free-energy surface.

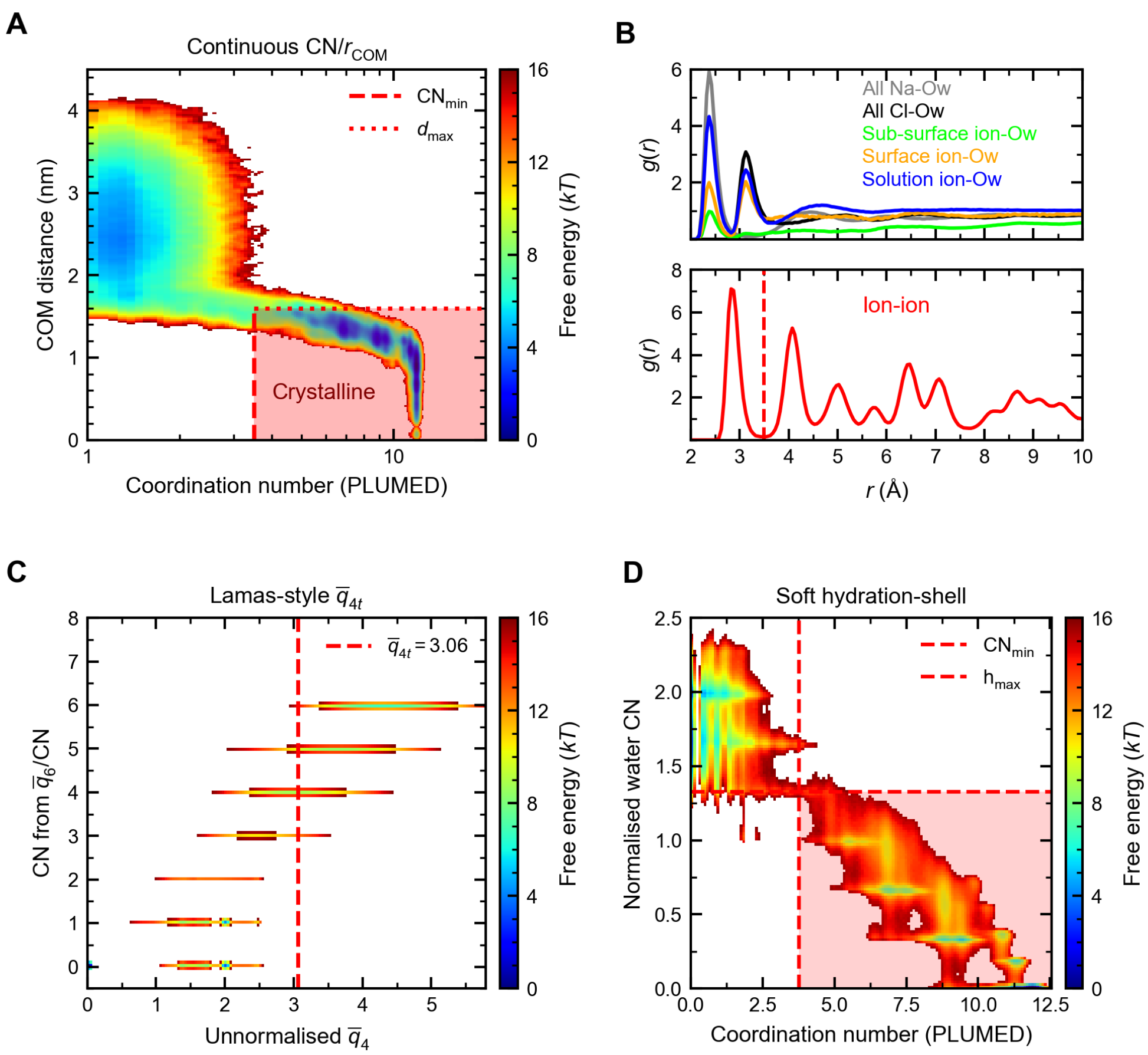

FIG. S1. **NaCl-8.** (A) Alternative two-dimensional clustering analysis gives $n_{ss} = 523$ for NaCl-8, which is statistically identical to $n_{ss} = 524$ obtained using the $\overline{q}_6$/CN-based route in the main text. (B) Radial distribution functions. (C) Correlation between the Lamas-style unnormalised $\overline{q}_4$, and the coordination number obtained from the $\overline{q}_6$/CN classification route. The dashed vertical line marks the threshold $\overline{q}_{4,t} = 3.06$, above which ions are classified as solid-like in this definition. (D) Two-dimensional free-energy surface constructed from the PLUMED-style continuous ion CN and the normalised soft water coordination number. The shaded region marks the low-hydration/high-CN crystalline assignment used in the definition.

S3. REAL-SOLUTION CORRECTION AND ACTIVITY-COEFFICIENT CONVERSION

Here we provide the derivation used to simplify the first derivative of the real-solution correction in the main text, together with the conversion between the molality-based activity model and the ionic mole-fraction notation used in the confined free-energy expression.

A. Gibbs–Duhem simplification

The non-ideal correction to the confined free energy is

$$\Delta F_{\mathrm{corr}}(n) = -nkT \ln\left(\frac{\gamma}{\gamma_{\mathrm{c}}}\right) + N_1 kT \ln\left(\frac{\gamma}{\gamma_0}\right) + N_2 kT \ln\left(\frac{\gamma_2}{\gamma_{2,0}}\right), \tag{S1}$$

where γ is the mean ionic activity coefficient and γ_2 is the activity coefficient of water in the finite parent phase. Differentiating with respect to n gives

$$\frac{d\beta\Delta F_{\mathrm{corr}}(n)}{dn} = -\ln\left(\frac{\gamma}{\gamma_{\mathrm{c}}}\right) + \left[(N_1 - n)\frac{d\ln\gamma}{dn} + N_2\frac{d\ln\gamma_2}{dn}\right]. \tag{S2}$$

The parent solution contains $(N_1 - n)$ ions and N_2 water molecules. At fixed temperature and pressure, the Gibbs–Duhem relation requires

$$(N_1 - n)d\mu_1 + N_2 d\mu_2 = 0. \tag{S3}$$

Using $\mu_1 = \mu_1^\circ + kT\ln(x\gamma)$ and $\mu_2 = \mu_2^\circ + kT\ln(x_2\gamma_2)$, this becomes

$$(N_1 - n)d\ln x + N_2 d\ln x_2 + (N_1 - n)d\ln\gamma + N_2 d\ln\gamma_2 = 0. \tag{S4}$$

For the finite parent-phase composition, $x = \frac{N_1 - n}{N_1 + N_2 - n}$ and $x_2 = \frac{N_2}{N_1 + N_2 - n}$, the ideal mole-fraction contribution cancels identically:

$$(N_1 - n)d\ln x + N_2 d\ln x_2 = 0. \tag{S5}$$

Therefore, the remaining activity-coefficient differentials satisfy

$$(N_1 - n)d\ln\gamma + N_2 d\ln\gamma_2 = 0. \tag{S6}$$

Along the cluster-size coordinate, this gives

$$(N_1 - n)\frac{d\ln\gamma}{dn} + N_2\frac{d\ln\gamma_2}{dn} = 0. \tag{S7}$$

Substituting Eq. S7 into Eq. S2 removes the bracketed term and yields

$$\frac{d\Delta F_{\mathrm{corr}}(n)}{dn} = -kT\ln\left(\frac{\gamma}{\gamma_{\mathrm{c}}}\right). \tag{S8}$$

B. Conversion from molality to ionic mole fraction

The activity-coefficient models used for aqueous NaCl are expressed on the molality scale, whereas the confined free-energy expression is written in terms of the ionic mole fraction. The ionic mole fraction is related to the molality m of NaCl formula units by

$$x = \frac{2m}{n_2 + 2m}, \tag{S9}$$

where $n_2 = 1/M_2 = 55.5\ \mathrm{mol\,kg^{-1}}$ is the number of moles of water per kilogram of solvent and $M_2 = 0.018\ \mathrm{kg\,mol^{-1}}$. Using the supersaturation definition $S = x/x_c$, the corresponding molality can be written as

$$m(S) = \frac{n_2 S x_c}{2(1 - S x_c)}. \tag{S10}$$

The mole-fraction and molality activity coefficients describe the same solute chemical potential. Therefore,

$$\ln x + \ln \gamma(x) = \ln m + \ln \gamma^m(m) + \text{constant}. \tag{S11}$$

Substituting Eq. S10 gives the mole-fraction activity coefficient at supersaturation S,

$$\ln \gamma(S) = \ln \gamma^m[m(S)] - \ln(1 - S x_c), \tag{S12}$$

up to an S-independent constant that cancels when referenced to coexistence. Hence, the real-solution thermodynamic driving force can be written as

$$\beta \Delta \mu(S) = \ln S + \ln \gamma(S) - \ln \gamma(S = 1). \tag{S13}$$

This expression is the activity correction used in the real-solution stationarity condition in the main text.

S4. REAL-SOLUTION APPROXIMATION

The Henry's law standard chemical potential of NaCl salt can be expressed as,

$$\beta\mu_{\mathrm{NaCl}}^{(m)} = \beta\mu_{\mathrm{NaCl}}^{\dagger,(m)} + 2\ln m + 2\ln\gamma_{\pm}^{m}(m) \tag{S14}$$

We can define the chemical potential per ion according to,

$$\beta\mu_{\pm}^{(m)} = \beta\mu_{\pm}^{\dagger,(m)} + \ln m + \ln\gamma_{\pm}^{m}(m) \tag{S15}$$

with the standard chemical potentials $\beta\mu_{\pm}^{\dagger,(m)} = \frac{1}{2}\beta\mu_{\mathrm{NaCl}}^{\dagger,(m)}$. This allows us to determine the chemical potential using the relationships between m and x:

$$\beta\mu_{\pm}^{x} = \beta\mu_{\pm}^{\dagger,x} + \ln x + \ln\gamma_{\pm}^{x}(x) \tag{S16}$$

Because Eq. S15 and Eq. S16 represent the same thermodynamic state ($\mu_{\pm}^{(m)} = \mu_{\pm}^{x}$), they can be equated to provide,

$$\beta\mu_{\pm}^{\dagger,(m)} + \ln m + \ln\gamma_{\pm}^{m}(m) = \beta\mu_{\pm}^{\dagger,x} + \ln x + \ln\gamma_{\pm}^{x}(x) \tag{S17}$$

By evaluating this equality at the limit of infinite dilution ($m \to 0$, where $x \to 0$ and both $\gamma_{\pm}^{m} \to 1$ and $\gamma_{\pm}^{x} \to 1$), we can obtain the exact relationship between the standard states. Using the mole fraction definition $x = \frac{2m}{n_2+2m}$, we find $\lim_{m\to 0}(x/m) = 2/n_2$. Therefore, the standard chemical potentials differ by a constant offset: $\beta\mu_{\pm}^{\dagger,(m)} - \beta\mu_{\pm}^{\dagger,x} = \ln(2/n_2)$. Substituting this standard-state difference back into Eq. S17 to equate the real states gives,

$$\ln\gamma_{\pm}^{x}(x) = \ln\gamma_{\pm}^{m}(m) + \ln\left(\frac{2m}{n_2 x}\right) \tag{S18}$$

Adopting $x = \frac{2m}{n_2+2m}$ provides the rigorous thermodynamic conversion between the two scales,

$$\ln\gamma_{\pm}^{x}(m) = \ln\gamma_{\pm}^{m}(m) + \ln\left(1 + \frac{2m}{n_2}\right) \tag{S19}$$

where $n_2 = 1/M_2 = 55.51$ mol kg^{-1}, and $M_2 = 0.018$ kg mol^{-1} is the molecular weight of water. Finally, with $S = x/x_c$ and $m(S) = \frac{n_2 S x_c}{2(1-Sx_c)}$, the activity coefficient on the mole fraction scale can be elegantly expressed directly as a function of supersaturation,

$$\ln \gamma_{\pm}^{x}(S) = \ln \gamma_{\pm}^{m}\left(m(S)\right) - \ln\left(1 - Sx_{\mathrm{c}}\right) \tag{S20}$$

For the ionic solute, we employ the Pitzer–Debye–Hückel model to approximate the mean ionic activity coefficient, $\gamma^m(m)$, defined on the molality scale m [2]:

$$\ln \gamma^{m}(m) = -A_{\phi}\left[\frac{\sqrt{m}}{1+b\sqrt{m}} + \frac{2}{b}\ln(1+b\sqrt{m})\right] + 2m\left[\beta_0 + \beta_1 g(\alpha\sqrt{m}) + C_{\phi}m\right] \tag{S21}$$

where $g(x) = 2[1-(1+x)e^{-x}]/x^2$.

As discussed in the main text, we employ two different approaches to depict the excess solute and solvent chemical potentials arising from solution non-idealities.

The first route is a thermodynamically consistent approach that utilizes a single set of Pitzer–Debye–Hückel parameters. By enforcing the Gibbs–Duhem relation, the osmotic coefficient $\phi(m)$ is analytically linked to $\ln\gamma^m$ [2]. In electrolyte thermodynamics, the activity of the solvent (water), a_2, can be directly computed from this osmotic coefficient via $\ln a_2(m) = -\nu m M_2 \phi(m)$, where $\phi(m)$ is given by:

$$\phi(m) = 1 - \frac{A_{\phi}\sqrt{m}}{1+b\sqrt{m}} + 2m[\beta_0 + \beta_1 f(\alpha\sqrt{m})] + 2C_{\phi}m^2 \tag{S22}$$

with $f(x) = 2[1-(1+x+\frac{1}{2}x^2)e^{-x}]/x^3$.

To obtain the Pitzer–Debye–Hückel parameters, we fix $\{A_{\phi} = 0.392, \alpha = 2.0, b = 1.2\}$ [2], and $\{\beta_0, \beta_1, C_{\phi}\}$ are then determined by fitting to the NIST data for solvent activity, $\{\ln a_2, m\}$, in the NaCl-H_2O system at 25 °C [3]. This is achieved by resolving the following least-squares minimisation problem:

$$\min_{\{\beta_0,\beta_1,C_{\phi}\}} \sum_{i=1}^{N_{\mathrm{data}}} \left[\ln a_2^{\mathrm{model}}(m_i;\, A_{\phi}, b, \alpha, \beta_0, \beta_1, C_{\phi}) - \ln a_2^{\mathrm{NIST}}(m_i)\right]^2 \tag{S23}$$

where N_{data} is the number of $\{\ln a_2, m\}$ data points. By using these solvent-fitted parameters to analytically compute $\ln\gamma^m(m)$ via Eq. S21, thermodynamic consistency between the solute and solvent is retained.

The second route relaxes the Gibbs–Duhem relation to optimise empirical accuracy for both the solute and solvent. In this decoupled approach, two independent sets of Pitzer–Debye–Hückel parameters are utilized: one for the solute ($\{\beta_{0,\mathrm{ion}}, \beta_{1,\mathrm{ion}}, C_{\phi,\mathrm{ion}}\}$) and one for the solvent ($\{\beta_{0,\mathrm{water}}, \beta_{1,\mathrm{water}}, C_{\phi,\mathrm{water}}\}$). While the universal constants $\{A_{\phi} =$

$0.392, \alpha = 2.0, b = 1.2\}$ remain fixed [2], the interaction parameters are obtained by simultaneously resolving two independent least-squares minimisation problems against the evaluated NIST data [3] at 25 °C.

For the solvent, the parameters are fitted to the NIST water activity data exactly as in Eq. S23:

$$\min_{\{\beta_{0,2},\beta_{1,2},C_{\phi,2}\}} \sum_{i=1}^{N_{\text{data}}} \left[\ln a_2^{\text{model}}(m_i;\, A_\phi, b, \alpha, \beta_{0,2}, \beta_{1,2}, C_{\phi,2}) - \ln a_2^{\text{NIST}}(m_i)\right]^2 \tag{S24}$$

For the solute, the independent ion parameters are fitted directly to the NIST mean ionic activity coefficient data, $\{\ln \gamma^m, m\}$:

$$\min_{\{\beta_{0,1},\beta_{1,1},C_{\phi,1}\}} \sum_{i=1}^{N_{\text{data}}} \left[\ln \gamma^{m,\text{model}}(m_i;\, A_\phi, b, \alpha, \beta_{0,1}, \beta_{1,1}, C_{\phi,1}) - \ln \gamma^{m,\text{NIST}}(m_i)\right]^2 \tag{S25}$$

By utilizing the independent parameter sets derived from Eq. S24 and Eq. S25 within Eq. S22 and Eq. S21 respectively, this dual-parameter model traces the empirical activity curves of both components.

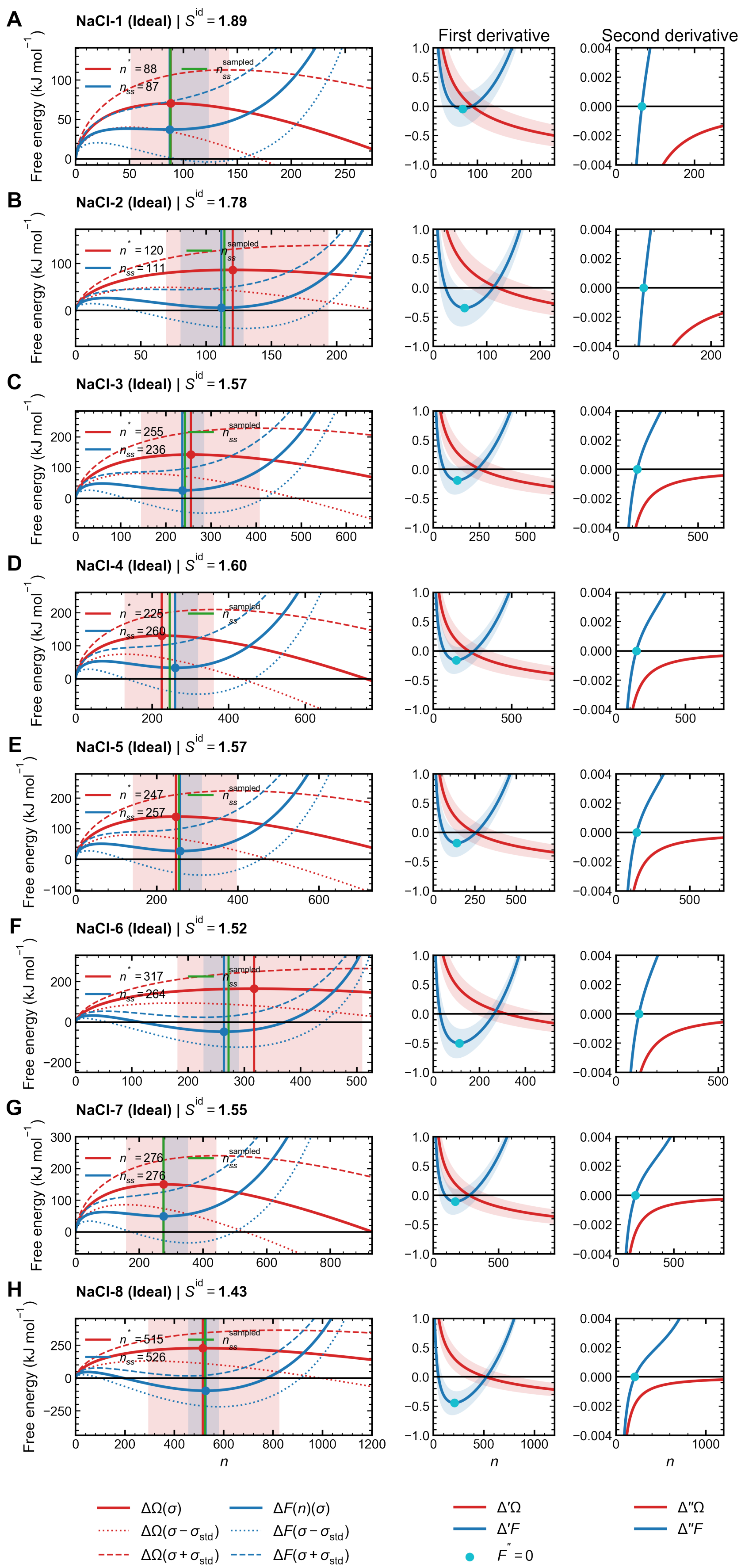
A NaCl-1 (Ideal) | S^{id} = 1.89
B NaCl-2 (Ideal) | S^{id} = 1.78
C NaCl-3 (Ideal) | S^{id} = 1.57
D NaCl-4 (Ideal) | S^{id} = 1.60
E NaCl-5 (Ideal) | S^{id} = 1.57
F NaCl-6 (Ideal) | S^{id} = 1.52
G NaCl-7 (Ideal) | S^{id} = 1.55
H NaCl-8 (Ideal) | S^{id} = 1.43
First derivative
Second derivative
Free energy (kJ mol^{-1})
n
$\Delta\Omega(\sigma)$
$\Delta\Omega(\sigma-\sigma_{std})$
$\Delta\Omega(\sigma+\sigma_{std})$
$\Delta F(n)(\sigma)$
$\Delta F(\sigma-\sigma_{std})$
$\Delta F(\sigma+\sigma_{std})$
$\Delta'\Omega$
$\Delta'F$
$F''=0$
$\Delta''\Omega$
$\Delta''F$

FIG. S2. Critical-cluster Equivalence Mapping for ideal solution, from NaCl-1 to NaCl-8 (Table II).

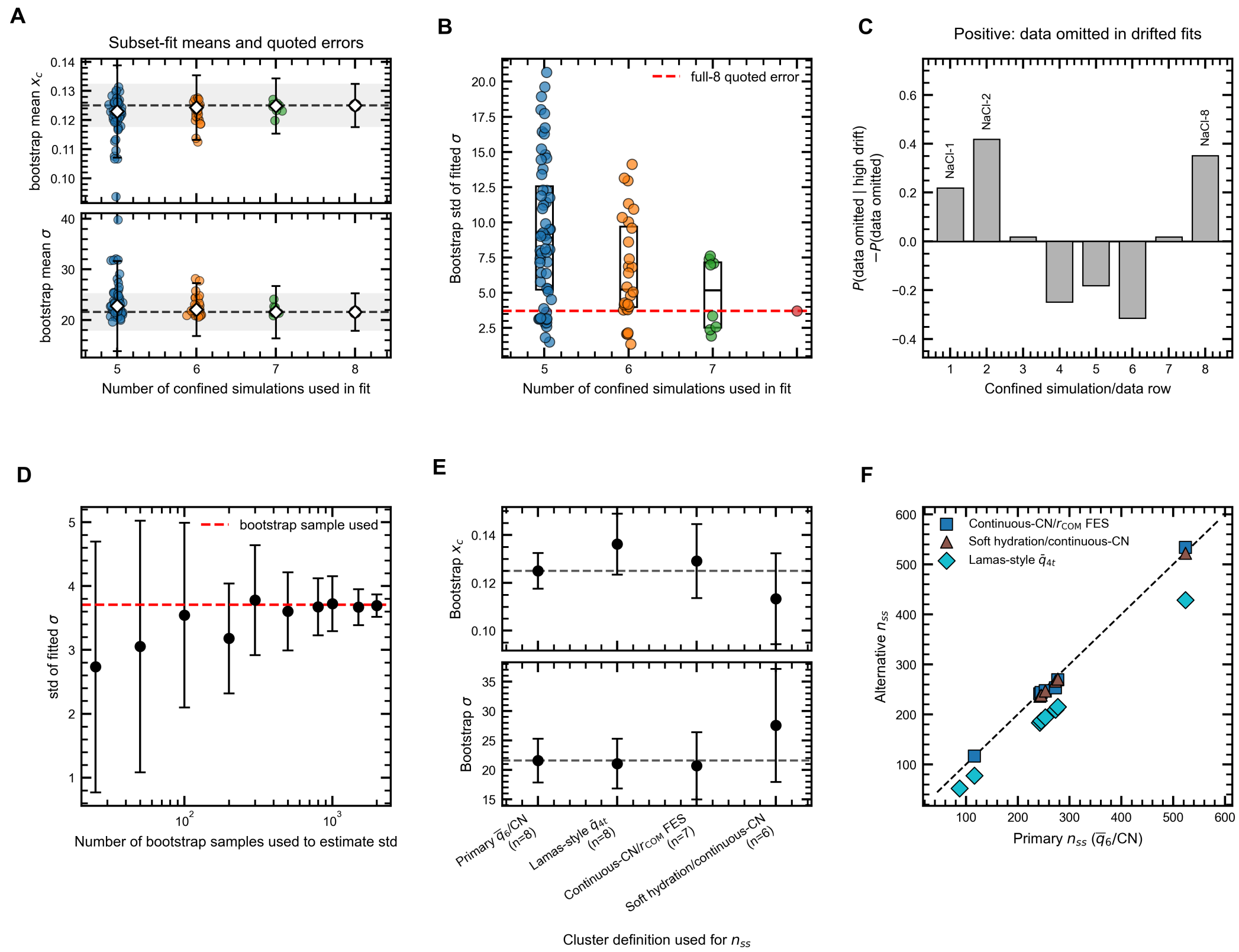

(Caption for this figure is on the following page)

FIG. S3. **Sensitivity of the ideal-solution NaCl residual fit to the number and choice of confined simulations, bootstrap statistics, and cluster definition.** (A) Bootstrap mean fitted coexistence concentration x_c and surface tension σ obtained from all 5-, 6-, 7-, and 8-row subsets of the primary $\bar{q}_6$/CN confined-simulation data. Open diamonds show the median subset result, with error bars corresponding to the median bootstrap standard deviation. The dashed line and shaded band indicate the full-8 bootstrap mean and one-standard-deviation interval. (B) Distribution of the bootstrap standard deviation of the fitted surface tension, σ_{std}, across all confined simulation subsets of a given size M. For each subset (all $\binom{8}{M}$ combinations of the eight NaCl systems: $\binom{8}{5} = 56$, $\binom{8}{6} = 28$, $\binom{8}{7} = 8$, and the single full-eight fit at $M = 8$), σ is fitted by bootstrap and its standard deviation recorded; each coloured point is one such subset. . At each M, the box spans the middle half of the subset values, and the line within it marks their median, while the coloured points show each individual subset. The red dashed line is the quoted full-eight uncertainty, $\sigma_{\text{std}}^{M=8}$. As M decreases, both the median and the spread of σ_{std} rise above the full-eight value, showing that fewer confined simulations give a larger and more variable σ uncertainty; this converges toward $\sigma_{\text{std}}^{M=8}$ as $M \to 8$.

FIG. S3. (C) Sensitivity of the subset fits. For each confined-simulation row, the plotted value is the frequency with which that data is absent from high-drift subset fits, minus the frequency with which it is absent across all subset fits. Here, high-drift subset fits are defined as those for which the fitted surface tension deviates from the full-8 result by at least one full-8 bootstrap standard deviation, i.e. $|\sigma_z| \geq 1$, with $\sigma_z = (\langle\sigma\rangle_{\text{subset}} - \langle\sigma\rangle_{\text{full,8}})/\text{std}(\sigma)_{\text{full,8}}$. Positive values, therefore, indicate data points whose omission is disproportionately associated with subset fits that yield σ values farthest from the full-8 result. For example, NaCl-2 is omitted in 11 of the 15 high-drift subset fits. Across all possible 5-, 6-, and 7-data subsets of the eight confined simulations, any given data is omitted in 29 of 92 subsets, giving a baseline omission probability of 29/92. Therefore, the value plotted for NaCl-2 is $11/15 - 29/92 = 0.42$. This positive value means that NaCl-2 is omitted much more frequently among the high-drift fits, so its omission is disproportionately associated with σ deviating from the full-8 result. Negative values have the opposite interpretation: those rows are omitted less often than expected among the high-drift fits, meaning that the drifted fits usually retain them. (D) Convergence of the estimated bootstrap standard deviation of the fitted surface tension, as a function of the number of bootstrap samples used to estimate it. Points show the mean estimate from repeated bootstrap draws of the indicated size, and error bars show the scatter of this estimate across repeats. The red dashed line marks the bootstrap sample size used in the main analysis. (E) Fitted coexistence concentration (x_c) and surface tension (σ) obtained using different definitions of the steady-state cluster size (n_{ss}). The dashed grey lines indicate the primary $\bar{q}_6$/CN result. Error bars denote bootstrap uncertainties. (F) Comparison of the alternative n_{ss} values with the primary $\bar{q}_6$/CN values. The dashed line indicates equality. The continuous-CN–r_{COM} and soft-hydration-shell definitions remain close to the primary definition, whereas the Lamas-style $\bar{q}_{4t}$ criterion systematically yields smaller clusters. Despite these differences in absolute cluster size, the refitted x_c and σ remain close to the primary values within the estimated uncertainties.

In Fig. S3, the subset analysis separates two sources of uncertainty in the residual fit. The central values of x_c and σ obtained from smaller subsets remain close to the full-8 result, indicating that the fitted thermodynamic parameters are not determined by a single confined simulation. However, the bootstrap spread of σ is broader and more heterogeneous for 5- and 6-row fits, showing that the surface-tension uncertainty is more sensitive to the particular subset when fewer confined simulations are used. To identify which data rows

contribute most to this subset sensitivity, we define high-drift subset fits as those for which the fitted σ differs from the full-8 mean by at least one full-8 bootstrap standard deviation, using the top 20 of subset fits ranked by $|\sigma_z|$ if too few subsets meet this threshold. The omission diagnostic further identifies which data points are disproportionately absent from the fits of the most-drifting subset. Overall, using all eight confined simulations stabilises both the fitted values and the bootstrap uncertainty.

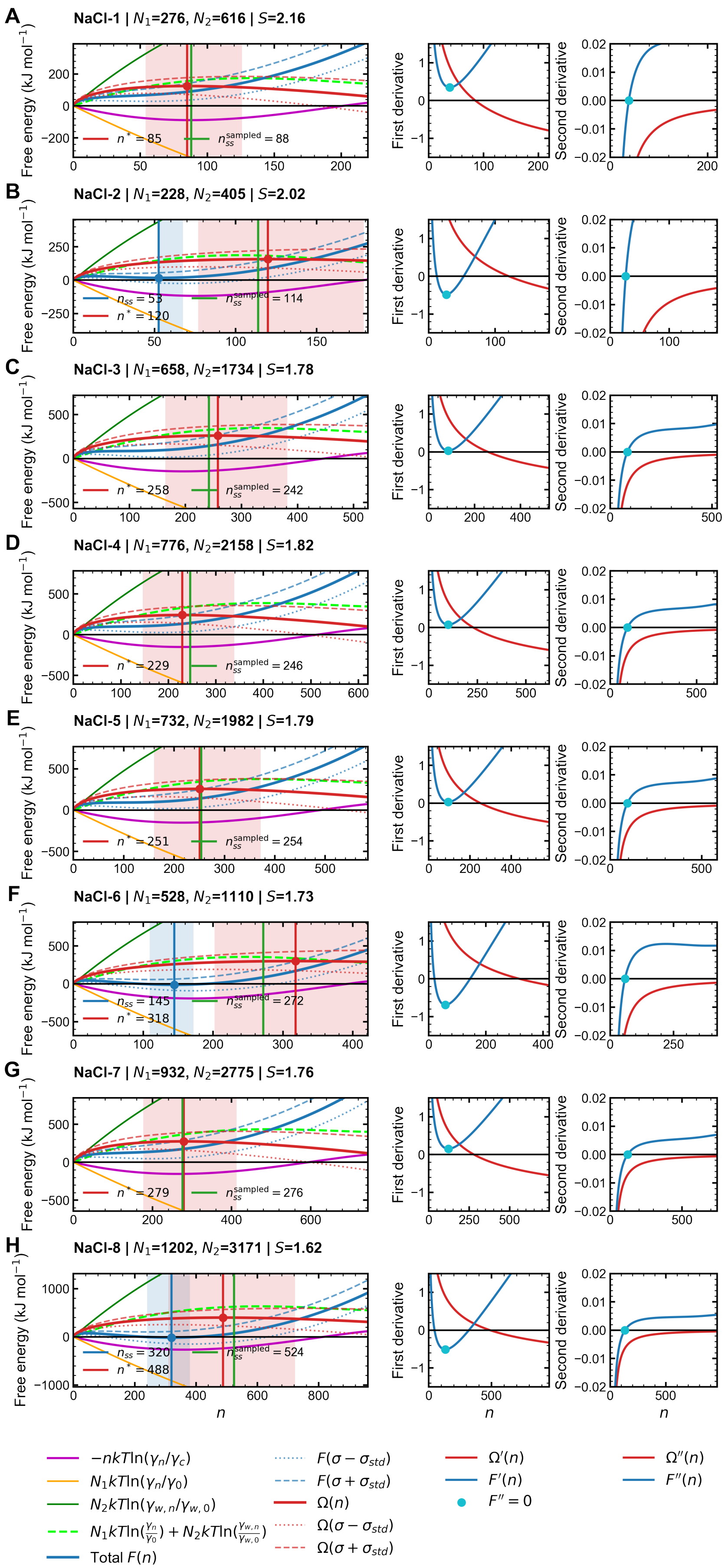
A
NaCl-1 | N_1=276, N_2=616 | S=2.16
$n^*=85$
$n_{ss}^{\text{sampled}}=88$
B
NaCl-2 | N_1=228, N_2=405 | S=2.02
$n_{ss}=53$
$n^*=120$
$n_{ss}^{\text{sampled}}=114$
C
NaCl-3 | N_1=658, N_2=1734 | S=1.78
$n^*=258$
$n_{ss}^{\text{sampled}}=242$
D
NaCl-4 | N_1=776, N_2=2158 | S=1.82
$n^*=229$
$n_{ss}^{\text{sampled}}=246$
E
NaCl-5 | N_1=732, N_2=1982 | S=1.79
$n^*=251$
$n_{ss}^{\text{sampled}}=254$
F
NaCl-6 | N_1=528, N_2=1110 | S=1.73
$n_{ss}=145$
$n^*=318$
$n_{ss}^{\text{sampled}}=272$
G
NaCl-7 | N_1=932, N_2=2775 | S=1.76
$n^*=279$
$n_{ss}^{\text{sampled}}=276$
H
NaCl-8 | N_1=1202, N_2=3171 | S=1.62
$n_{ss}=320$
$n^*=488$
$n_{ss}^{\text{sampled}}=524$
Free energy (kJ mol^{-1})
First derivative
Second derivative
n
$-nkT\ln(\gamma_n/\gamma_c)$
$N_1kT\ln(\gamma_n/\gamma_0)$
$N_2kT\ln(\gamma_{w,n}/\gamma_{w,0})$
$N_1kT\ln(\frac{\gamma_n}{\gamma_0}) + N_2kT\ln(\frac{\gamma_{w,n}}{\gamma_{w,0}})$
Total $F(n)$
$F(\sigma - \sigma_{std})$
$F(\sigma + \sigma_{std})$
$\Omega(n)$
$\Omega(\sigma - \sigma_{std})$
$\Omega(\sigma + \sigma_{std})$
$\Omega'(n)$
$F'(n)$
$F'' = 0$
$\Omega''(n)$
$F''(n)$

FIG. S4. **A1 activity model**. Critical Cluster Equivalence Principle for real solution fitted with NIST $\ln a_2$ data, from NaCl-1 to NaCl-8 (Table II).

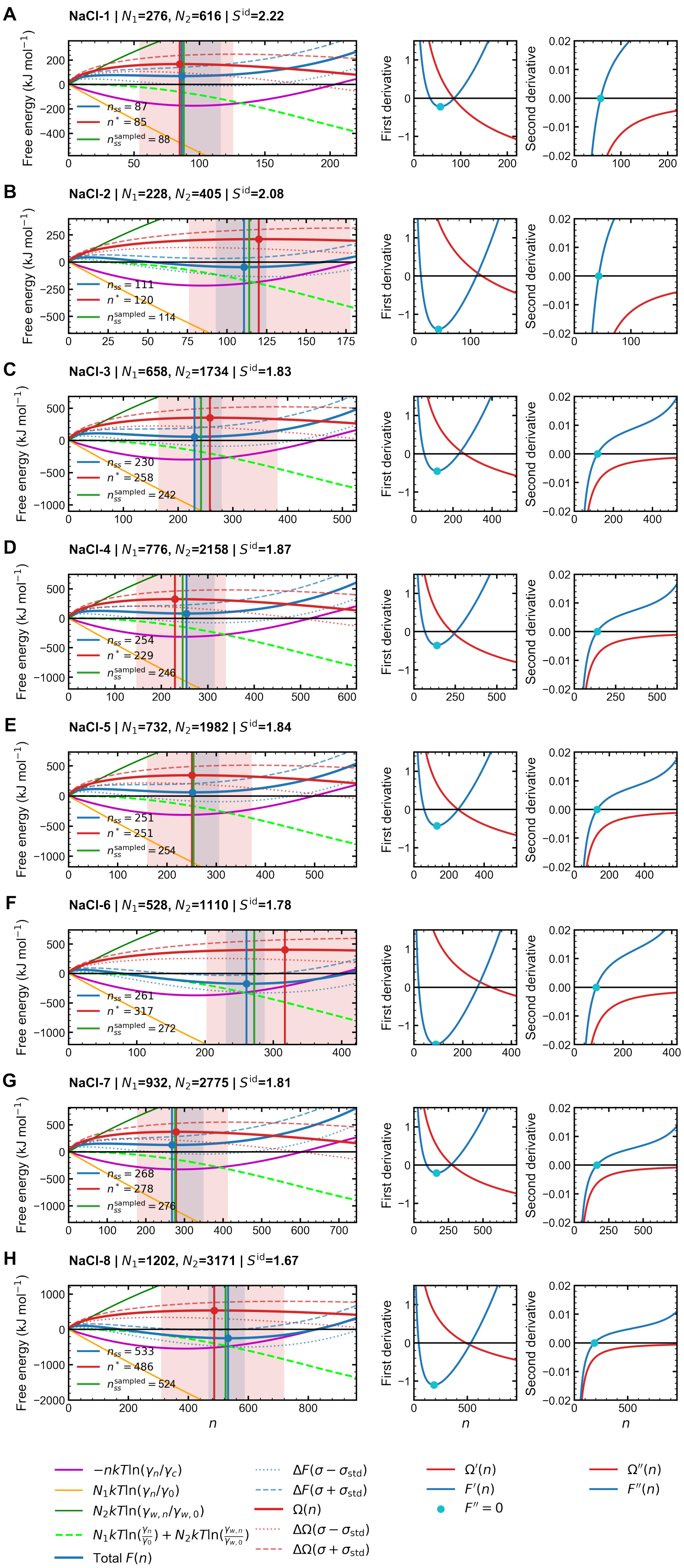
A
NaCl-1 | N1=276, N2=616 | Sid=2.22
nss = 87
n* = 85
nss sampled = 88
B
NaCl-2 | N1=228, N2=405 | Sid=2.08
nss = 111
n* = 120
nss sampled = 114
C
NaCl-3 | N1=658, N2=1734 | Sid=1.83
nss = 230
n* = 258
nss sampled = 242
D
NaCl-4 | N1=776, N2=2158 | Sid=1.87
nss = 254
n* = 229
nss sampled = 246
E
NaCl-5 | N1=732, N2=1982 | Sid=1.84
nss = 251
n* = 251
nss sampled = 254
F
NaCl-6 | N1=528, N2=1110 | Sid=1.78
nss = 261
n* = 317
nss sampled = 272
G
NaCl-7 | N1=932, N2=2775 | Sid=1.81
nss = 268
n* = 278
nss sampled = 276
H
NaCl-8 | N1=1202, N2=3171 | Sid=1.67
nss = 533
n* = 486
nss sampled = 524
Free energy (kJ mol−1)
First derivative
Second derivative
n
−nkT ln(γn/γc)
N1kT ln(γn/γ0)
N2kT ln(γw,n/γw,0)
N1kT ln(γn/γ0) + N2kT ln(γw,n/γw,0)
Total F(n)
ΔF(σ − σstd)
ΔF(σ + σstd)
Ω(n)
ΔΩ(σ − σstd)
ΔΩ(σ + σstd)
Ω′(n)
F′(n)
F″ = 0
Ω″(n)
F″(n)

FIG. S5. **A2 activity model**. Critical Cluster Equivalence Principle for real solution fitted with NIST $\ln a_2$ and $\ln \gamma^m$ data, from NaCl-1 to NaCl-8 (Table II).

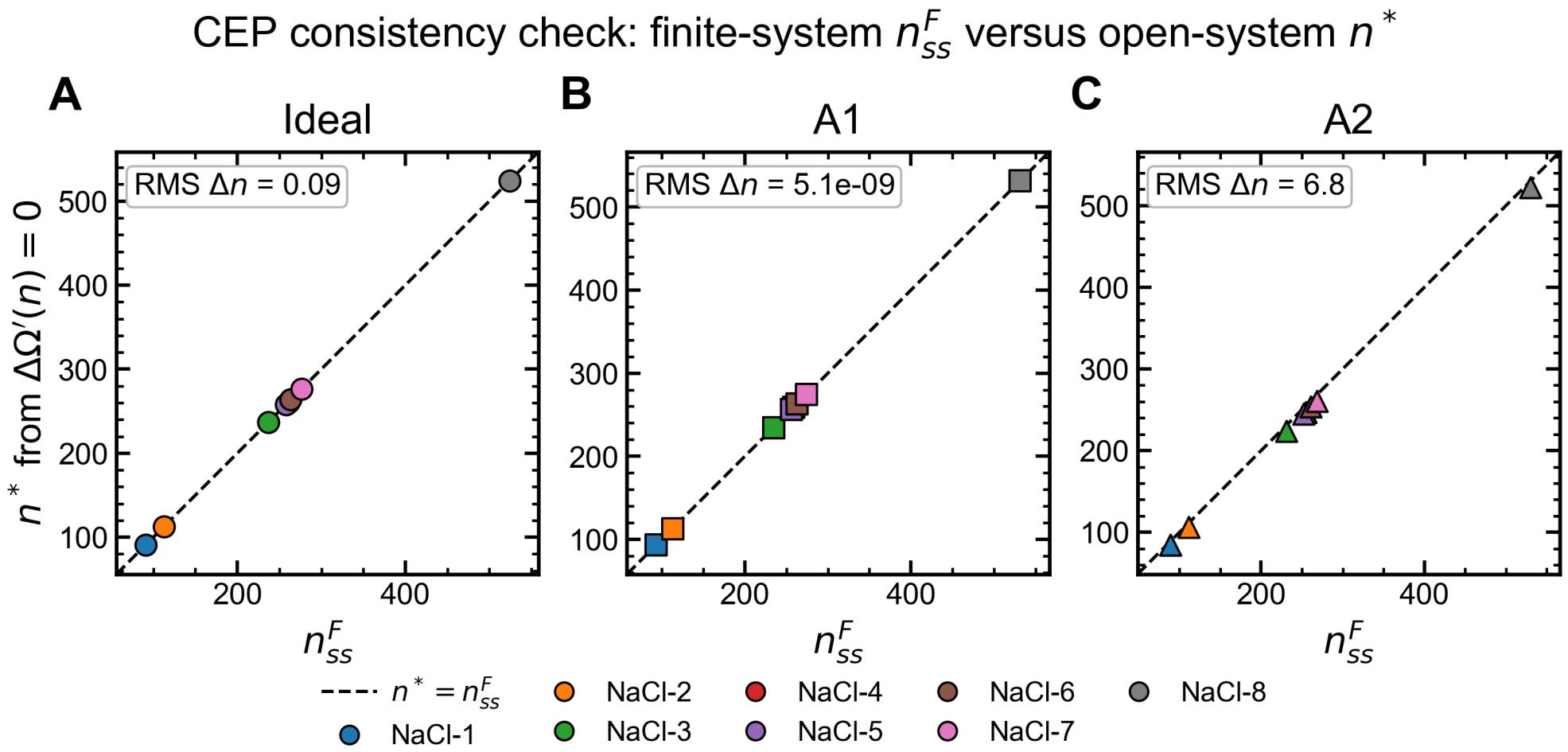

FIG. S6. **Stationary-point consistency check for the ideal, A1, and A2 NaCl free-energy models.** Comparison between the finite-system stationary point, $n_{\rm ss}^F$, obtained from the minimum of the confined Helmholtz free-energy profile $\Delta F(n)$, and the open-system critical size, n^*, obtained from the maximum of the corresponding osmotic free-energy profile $\Delta\Omega(n)$. Results are shown for (A) the ideal-solution model, (B) the A1 activity model, and (C) the A2 activity model. The dashed line indicates the strict CEP condition ($n^* = n_{\rm ss}^F$). Colours identify the eight confined NaCl simulations, NaCl-1 to NaCl-8. The reported RMS value is $\mathrm{RMS}_{\Delta n} = \sqrt{\langle (n^* - n_{\rm ss}^F)^2 \rangle}$, calculated over the eight systems. The ideal and A1 models satisfy the stationary-point mapping to within numerical precision or sub-ion deviations, whereas A2 shows a systematic offset between the two stationary points.

In Fig. S6, the strict stationary-point comparison separates the thermodynamic CEP mapping from the residual quality of the fitted gradient at the sampled $n_{\rm ss}^{\rm sampled}$. For the ideal-solution model, $n_{\rm ss}^F$ and n^* agree (within much less than one ion) across the eight confined simulations, confirming that the small apparent deviations in the derivative plots (Figs. S2, S4 and S5) arise from residual fitting not exactly equal to zero for each $n_{\rm ss}^{\rm sampled}$. The A1 activity model also preserves the stationary-point equivalence, consistent with its Gibbs–Duhem-constrained construction: because A1 links the solvent activity with the solute activity through the osmotic coefficient (SI Appendix, Section 3A), the parent-phase activity-coefficient gradient $[(N_1 - n)\,\mathrm{d}\ln\gamma/\mathrm{d}n + N_2\,\mathrm{d}\ln\gamma_2/\mathrm{d}n]$ cancels exactly, so the confined gradient $\Delta F/\mathrm{d}n$ reduces to the same functional form as the open gradient $\Delta\Omega/\mathrm{d}n$, and the two profiles share the same stationary point. The small ideal-solution RMS $\Delta n \approx 0.1$

ion is a grid-resolution effect in locating n^*, not a real deviation: the ideal and A1 mappings are both exact to within numerical precision. By contrast, A2 gives a systematic displacement of several ions between n_{ss}^F and n^*. This follows directly from relaxing the Gibbs–Duhem constraint: A2 fits the solute and solvent activities to two independent NIST datasets, $\{\ln \gamma^m, m\}$ and $\{\ln a_2, m\}$ without linking them, so the parent-phase gradient above no longer vanishes exactly (SI Appendix, Section 3A). This leaves a small uncancelled term in the confined gradient that has no counterpart in the open gradient; ΔF minimum and the Ω are therefore no longer constrained to coincide. The trade-off is that this additional empirical flexibility improves the local derivative balance at the sampled $n_{\mathrm{ss}}^{\mathrm{sampled}}$ (Figs. S7), at the cost of a small loss of strict closed/open stationary-point consistency.

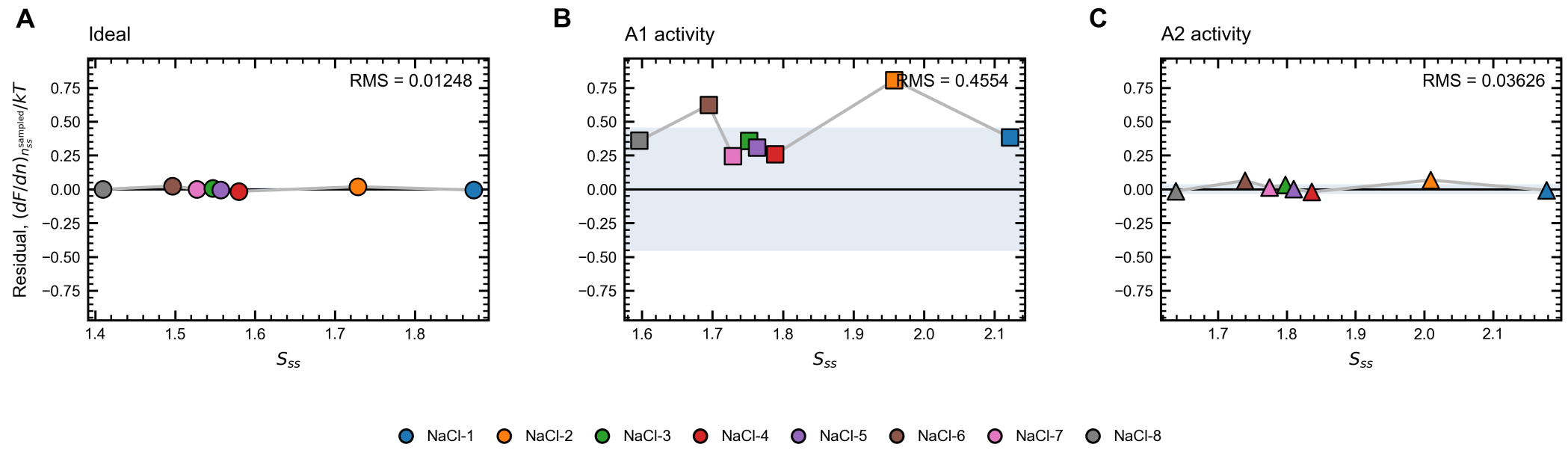

FIG. S7. **Stationarity residual diagnostics for the NaCl fits.** (A–C) Derivative residuals of, $r_i = (dF/dn)_{n_{ss,i}^{\text{sampled}}}/kT$, evaluated at the sampled confined-cluster size for each simulation using the ideal, A1 activity, and A2 activity models, respectively. A zero residual indicates that the sampled cluster size coincides exactly with the stationary point of the fitted closed-bath free-energy model. The shaded band indicates $\pm$ the root-mean-square (RMS) residual, $= \sqrt{\langle r_i^2 \rangle}$, where $r_i = (dF/dn)_{n_{ss,i}^{\text{sampled}}}$.

In Fig. S7, panels A-C quantify the residual diagnostics. In the thermodynamic formulation, non-ideality enters through the chemical potentials. The larger A1 residual reflects the more restrictive finite-system fitting caused by the chosen activity correction. Non-ideal activity terms differ between A1 and A2, so the two activity models enter the same CEP stationarity fit with different chemical-potential corrections. Once these activity curves are fixed, the residual fitting can only adjust the common coexistence concentration and surface tension, $\{x_c, \sigma\}$. Because the A1 activity correction is more constrained, the same shared x_c, σ fit must accommodate the finite-system data with less flexibility in the activity contribution. This gives a larger remaining stationary-point residual in panel B. By contrast, the A2 empirical activity description provides a more flexible representation over the sampled supersaturation range, leading to a smaller residual magnitude in panel C.

S5. IDEAL-SOLUTION AND REAL-SOLUTION COMPONENTS AS FUNCTIONS OF S_{NaCl} FOR COMPUTING J_{NaCl}

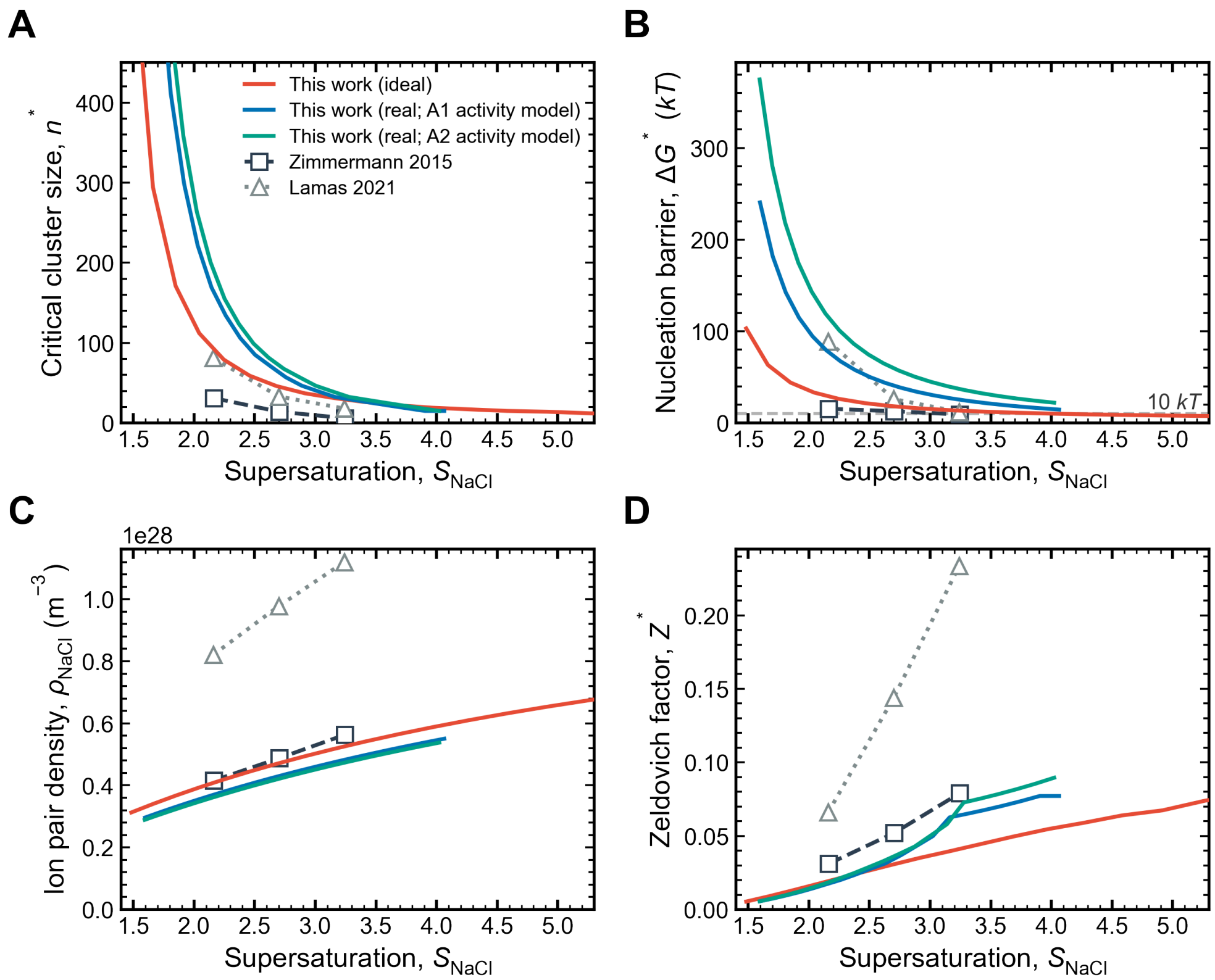

FIG. S8. (A) Critical nucleus size n^*. (B) Corresponding free energy barrier ΔG^*. (C) Molecular number density ρ_{NaCl}. (D) Zeldovich factor Z^*. Computational literature values [4, 5].

S6. CRITICAL-CLUSTER EQUIVALENCE MAPPING AND NUCLEATION RATES FOR ARGON

For a single-component system in a canonical (NVT) description, the Helmholtz free energy is the sum of contributions from the mother phase, the nucleus, and the interface,

$$F = F_m + F_n + F_{in}$$

Similar to the multicomponent case, using the equimolecular dividing surface to define properties of the interface, so that the nucleus contains n molecules and the mother phase contains $(N-n)$ molecules, the differentials of the three contributions are,

$$dF_m = -S_m dT - p dV_m + \mu d(N-n)$$

$$dF_n = -S_n dT - p_n dV_n + \mu_n dn$$

$$dF_{in} = -S_{in} dT + \sigma dA$$

where σ is the surface tension and A is the surface area of the crystal. For an incompressible nucleus, A and V_n depend only on the nucleus size, therefore,

$$dF = -SdT - pdV + \mu dN + \left[(p-p_n)\frac{dV_n}{dn} + (\mu_n - \mu) + \sigma\frac{dA}{dn}\right]dn \tag{S26}$$

where the quantity in square brackets is the derivative of the thermodynamic potential with respect to the reaction coordinate n.

Converting the fundamental relation Eq. S26 to a different ensemble involves taking a Legendre transform with respect to the corresponding thermodynamic variables. For example, going from the canonical (NVT) to the isothermal-isobaric (NpT), we would obtain,

$$\begin{aligned} dG &= d(F+pV) \\ &= -SdT + Vdp + \mu dN + \left[(p-p_n)\overline{v}_n + (\mu_n-\mu) + \sigma\frac{dA}{dn}\right]dn \end{aligned} \tag{S27}$$

where $\overline{v}_n = \frac{dV_n}{dn}$ is the molecular volume of the nucleus. In the case of argon vapour-liquid transition, $\overline{v}_n$ is thus the molecular volume of the liquid phase. This illustrates that the derivative of the thermodynamic potential with respect to the reaction coordinate (in this case,se n) is the same regardless of the thermodynamic ensemble [6], as long as the relevant natural variables are held constant. For example,

$$\left(\frac{\partial F}{\partial n}\right)_{N,V,T} = \left(\frac{\partial G}{\partial n}\right)_{N,p,T} \tag{S28}$$

A consequence of Eq. S28 is that a stationary point in a plot of the thermodynamic potential vs. n in one ensemble must also be a stationary point in any other ensemble (cluster equivalence), again with the caveat that the variables held constant are different.

The equations above justify the original cluster equivalence principle, or Free-energy reconstruction from stable clusters (FRESC) of Reguera *et al.* [7]. In the case of a single-component system, holding the temperature and pressure constant is equivalent to holding the chemical potential constant due to the Gibbs-Duhem relation. Therefore, the NpT representation is equivalent to an open system (this is the only case where we can hold both N and μ constant), and its stationary point occurs at the same value of n as in the closed system (NVT). For a mixture, however, holding p and T is not enough to represent an open system, since there is more than one chemical potential (see the multicomponent case in the main text).

For argon, based on CNT, the modified-liquid-droplet (MLD) model expresses the work required to form a cluster of size n in a closed NVT system [8, 9],

$$\Delta F(n) = -nkT \ln \frac{\rho}{\rho_{\mathrm{c}}} + 4\pi\sigma \left(\frac{3n\overline{v}_n}{4\pi}\right)^{2/3} + nkT(1 - \overline{v}_n \rho_{\mathrm{c}}) + NkT \ln \frac{\rho}{\rho_0} \tag{S29}$$

where ρ is the actual vapour density of the remaining $N - n$ vapour particles and ρ_0 corresponds to n=0.

To address the confinement effects, Reguera *et al.* combined canonical thermodynamics with the Young–Laplace relation for pressure differences across curved interfaces, deriving an expression for the equilibrium vapour density in confined systems[8, 9],

$$\rho_{\mathrm{c},i} = \rho_{\mathrm{c}} \exp\left[\frac{2\beta\sigma\overline{v}_n}{\sqrt[3]{3n_{ss,i}v_l/(4\pi)}}\right] \tag{S30}$$

where $n_{ss,i}$ denotes the number of particles in the equilibrium steady-state cluster in the system i, and the denominator defines its corresponding radius $r_{ss,i}$. In previous work [10], we showed that Eq. S30 can be used to determine key thermodynamic parameters (equilibrium density, ρ_{c}, and surface tension, σ) of bimolecular condensates modelled with coarse-grained force fields, by performing M independent NVT simulations and solving the following optimisation problem,

$$\min_{\rho_{\mathrm{c}},\sigma} \sum_{i=1}^{M} \left[\rho_{\mathrm{c},i} - \frac{N_i - n_{ss,i}}{V_i - n_{ss,i} v_l} \right]^2 \tag{S31}$$

where N_i and V_i are the total number of particles and the system volume, respectively, in simulation i. With ρ_{c} and σ determined, the free energy of forming a cluster of size n in both open (NpT) and confined (NVT) ensembles can be evaluated.

CNT generally assumes a curvature-independent surface tension, as expressed in Eq. S31. However, nanoscale nuclei are finite and spherical, leading to deviations from the planar limit. These curvature effects are captured by Tolman's equation [11, 12], which relates the surface tension σ_T to the critical radius r^*,

$$\sigma_{\mathrm{Tolman}} = \frac{\sigma_\infty}{1 + 2\delta / r^*} \tag{S32}$$

where σ_∞ denotes the planar surface tension, and δ, the Tolman length, quantifies the offset between the surface of tension and the equimolar surface, the theoretical dividing surface where the number of molecules from each phase is equal. While δ is formally defined in the limit of a planar interface, it can be reasonably approximated as a constant for droplets with $r \gg \delta$ [11, 13].

Figures S9 A-F show our results obtained for argon at T =80.7 K. Figures S10 and S11 illustrate CEP for argon at T =80.7 K for all simulated systems.

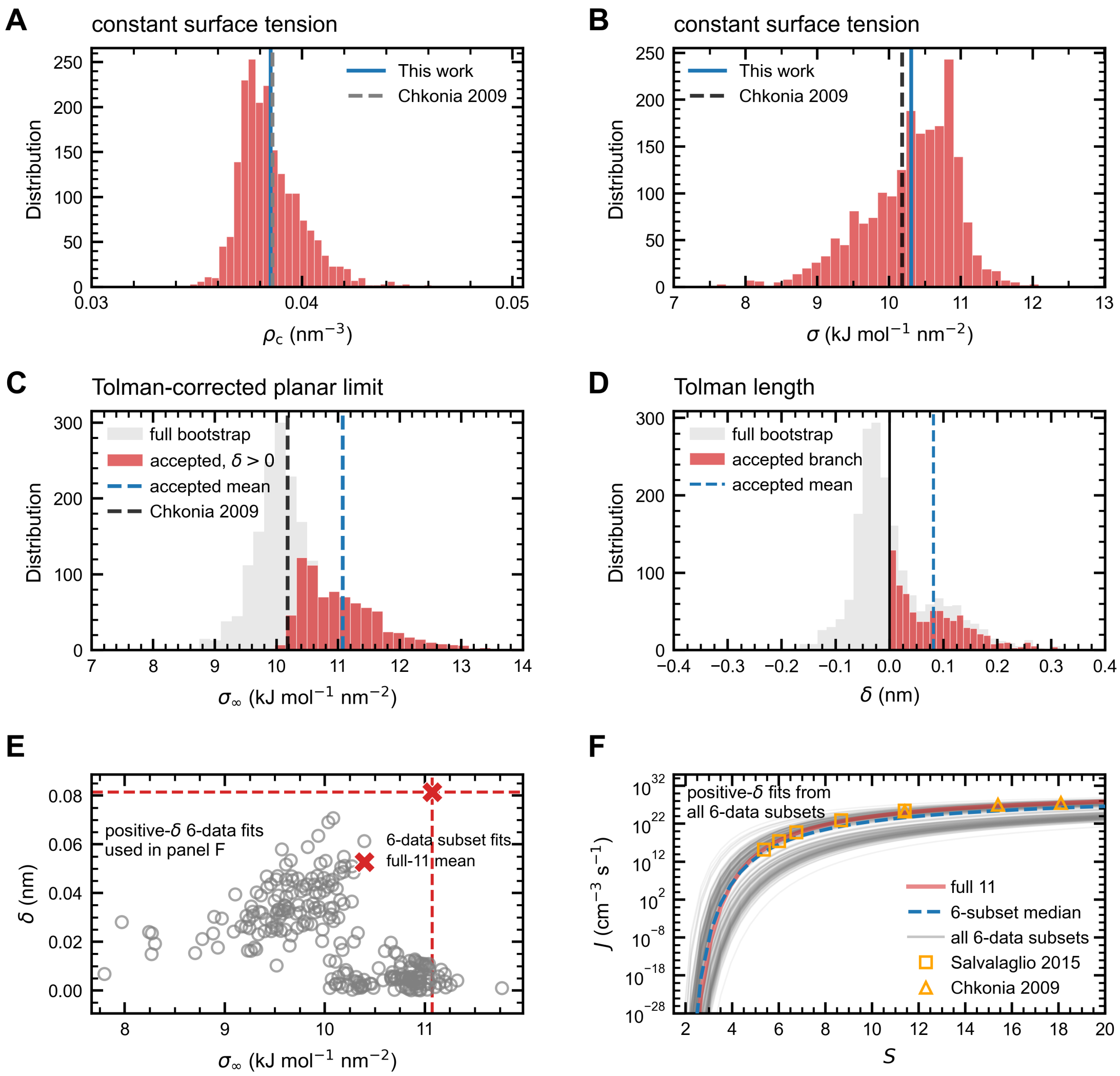

(Caption for this figure is on the following page)

FIG. S9. **Bootstrap and subset diagnostics for the Tolman-corrected argon fit.** (A) Bootstrap distribution of the coexistence vapour number density, ρ_c, obtained from the constant-surface-tension fit, compared with the Chkonia reference value. (B) Bootstrap distribution of the constant surface tension, σ, compared with the Chkonia reference value. (C) Bootstrap distribution of the Tolman planar-limit surface tension σ_∞. The grey shadow shows the full unconstrained bootstrap distribution, whereas the red histogram shows the accepted positive-δ branch used in the final Tolman analysis. (D) Bootstrap distribution of the Tolman length, δ. The grey shadow shows the full unconstrained bootstrap distribution, including the discarded negative-δ branch, whereas the red histogram shows the accepted positive-δ results. (E) Correlation between the fitted σ_∞ and the positive-δ Tolman parameters from all six-data subset fits. Grey open circles show the subset fits used for the grey rate curves in F, while the red marker show the full-eleven-data mean. (F) Tolman-corrected rate curves obtained from the positive-δ fits to all six-data subsets. Grey curves show individual subset predictions, the red curve shows the full-eleven-data prediction, and the blue dashed curve shows the median of the subset predictions evaluated in $\log_{10} J$ space at each supersaturation.

In Figs. S9C and D, the bootstrap diagnostics show that the Tolman fit is not unique without a physical selection: the unconstrained bootstrap distribution contains substantial negative-δ results, whereas the final reported Tolman prediction uses the accepted positive-δ group. In Fig. S9E, the six-data subset analysis further illustrates that reducing the number of confined simulations increases the variability of the fitted Tolman parameters and hence the predicted rate curves. In Figs. S9F, the subset analysis also shows how sensitive the Tolman-corrected rate prediction is to the finite set of confined simulations used in the fit. For each accepted positive-δ six-data subset k ($k = 1, \ldots, N_{\text{sub}}$), we evaluate a Tolman-corrected rate curve $J_k(S)$ on the same supersaturation as the full eleven-data prediction. At each point S_i we sort the N_{sub} subset values $\log_{10} J_k(S_i)$ and take the middle one (the median):

$$\tilde{y}_i = \operatorname*{median}_k \log_{10} J_k(S_i), \qquad J_{\text{med}}(S_i) = 10^{\tilde{y}_i}. \tag{S33}$$

The grey curves in Figs. S9F show the individual subset predictions. The accepted six-data subsets span a wider range of σ_∞ and δ than the full eleven-data fit, giving this spread. The full eleven-data prediction lies within the envelope but is smoother and less sensitive

to the omission of individual points, supporting the use of the more confined-equilibrium dataset for the reported argon rate.

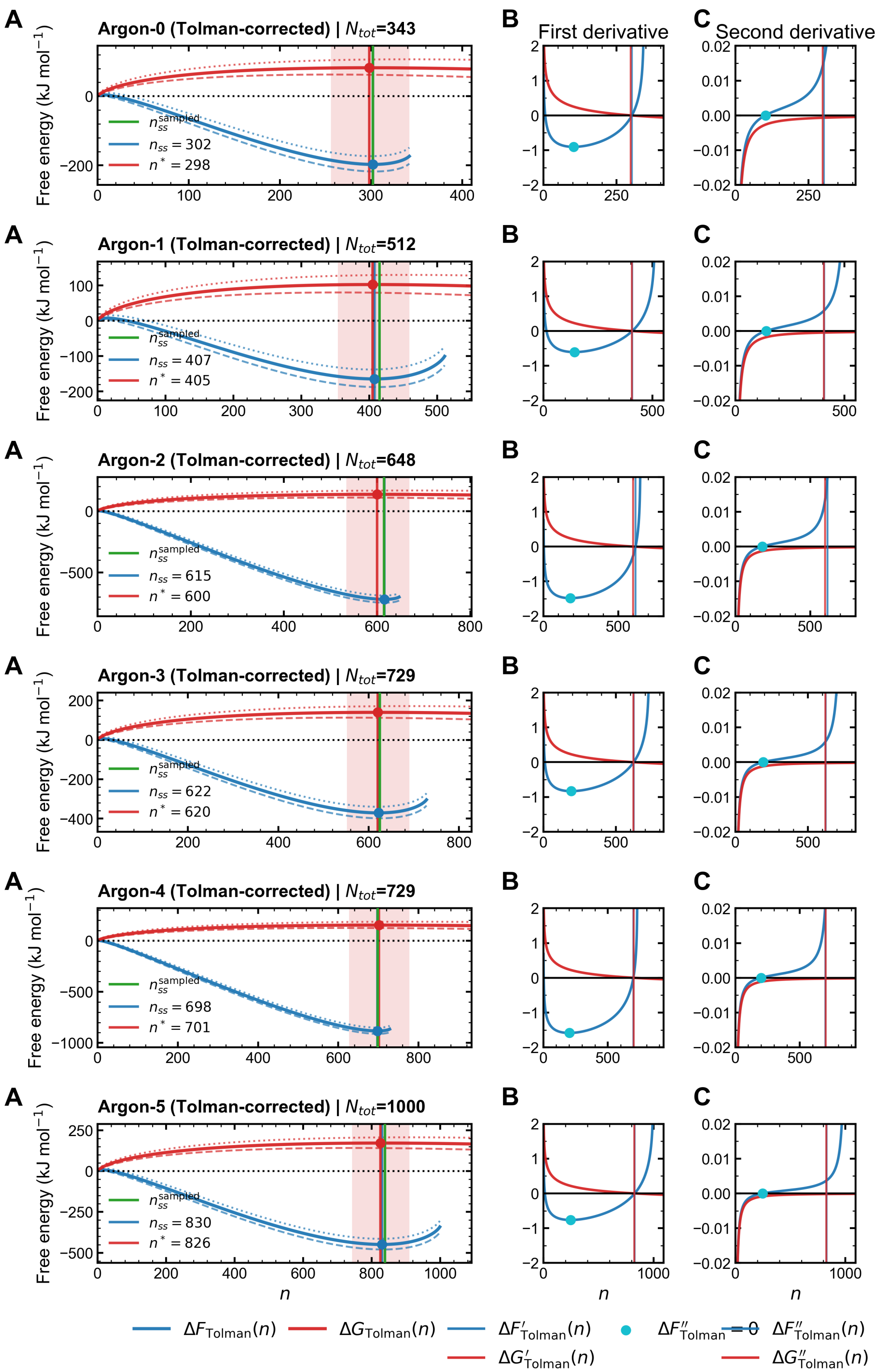

(Caption for this figure is on the following page)

FIG. S10. CEP with Tolman-correction for argon simulated at T=80.7 K.

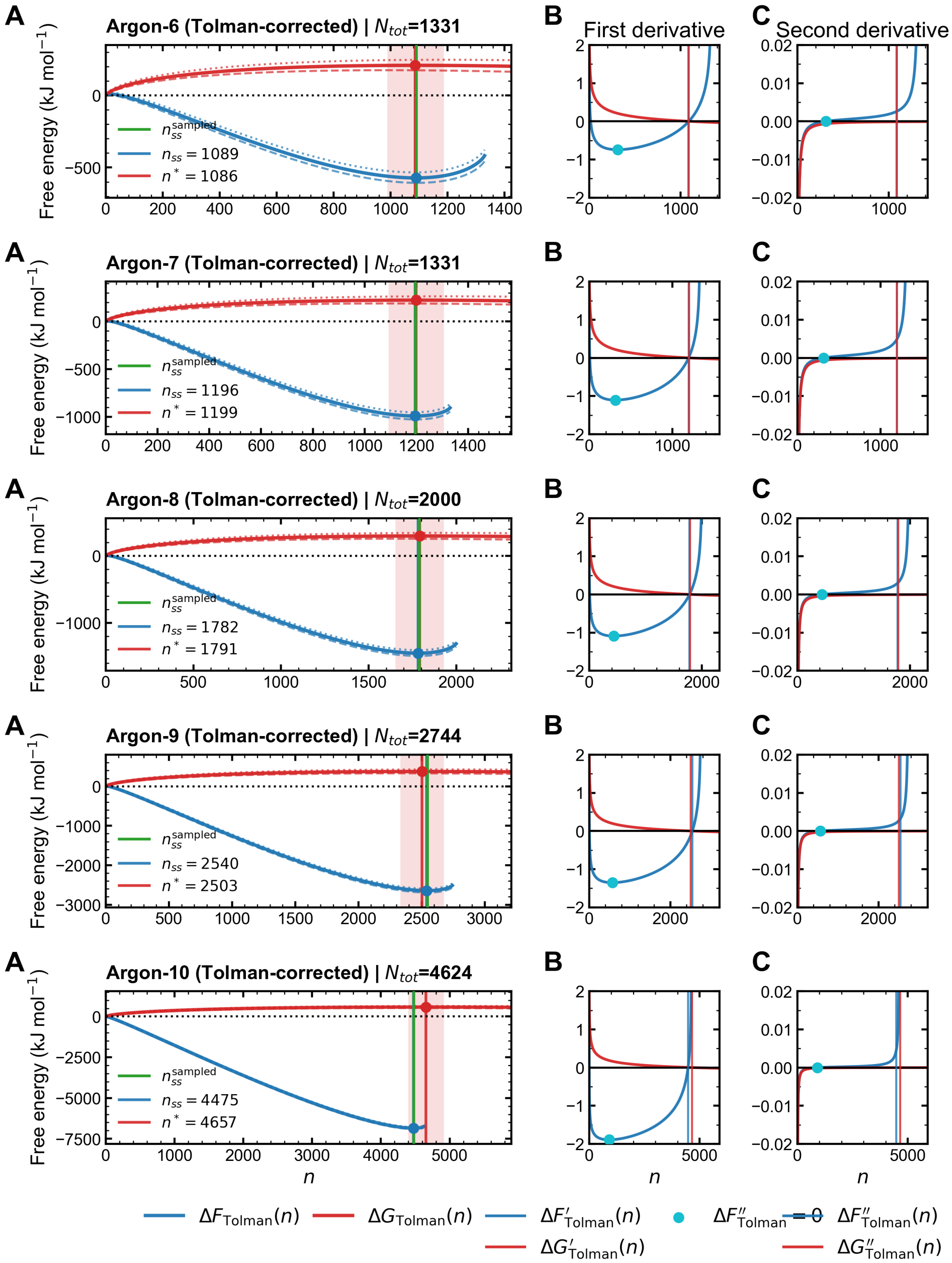

Tolman – corrected Equations used in this figure :

$$\sigma_{\mathrm{Tolman}}(r) = \frac{\sigma_\infty}{1 + 2\delta/r} \qquad r = \left(\frac{3\overline{v}_n n}{4\pi}\right)^{1/3}$$

$$\Delta F_{\mathrm{Tolman}}(n) = -nkT\ln\left(\frac{\rho}{\rho_c}\right) + \frac{\sigma_\infty}{1+2\delta/r}4\pi r^2 + nkT(1-\overline{v}_n\rho_c) + NkT\ln\left(\frac{\rho}{\rho_0}\right)$$

$$\Delta G_{\mathrm{Tolman}}(n) = -nkT\ln\left(\frac{\rho^*}{\rho_c}\right) + \frac{\sigma_\infty}{1+2\delta/r}4\pi r^2$$

$$\Delta F'_{\mathrm{Tolman}}(n) = \overline{v}_n\sigma_\infty\left[\frac{2}{r+2\delta} + \frac{2\delta}{(r+2\delta)^2}\right] + \overline{v}_n kT(\rho-\rho_c) - kT\ln\left(\frac{\rho}{\rho_c}\right)$$

$$\Delta G'_{\mathrm{Tolman}}(n) = \overline{v}_n\sigma_\infty\left[\frac{2}{r+2\delta} + \frac{2\delta}{(r+2\delta)^2}\right] - kT\ln\left(\frac{\rho^*}{\rho_c}\right)$$

$$\Delta F''_{\mathrm{Tolman}}(n) = \frac{\overline{v}_n^2}{4\pi r^2}\sigma_\infty\left[-\frac{2}{(r+2\delta)^2} - \frac{4\delta}{(r+2\delta)^3}\right] + \frac{kT}{N-n}(1-\rho\overline{v}_n)^2$$

$$\Delta G''_{\mathrm{Tolman}}(n) = \frac{\overline{v}_n^2}{4\pi r^2}\sigma_\infty\left[-\frac{2}{(r+2\delta)^2} - \frac{4\delta}{(r+2\delta)^3}\right]$$

FIG. S11. CEP with Tolman-correction for argon simulated at T=80.7 K.

In order to elucidate that diffusion is not sufficient to account for f^- in liquid argon droplets, we investigate the self-diffusion of argon particles contained within the liquid droplets. We calculate the mean-squared displacement (MSD) for a representative subset of 20 particles per system, using trajectories corresponding to their longest continuous residence within the droplet. From these trajectories, we derive the system-specific, steady-state internal diffusion coefficient $D_{ss} = \langle D_j \rangle_{j=1}^{20}$. Subsequently, we compute the characteristic diffusive timescale for a particle to diffuse from the droplet center to the surface at radial distance r_{ss}. This is expressed mathematically as $r_{ss}^2/[6D_{ss}]$. This quantity may be considered as the residence time for a particle originating from the center of the droplet. For a particle originating at the surface, the effective path length is roughly doubled, resulting in a characteristic diffusion time of $r_{ss}^2/[3D_{ss}]$. Moreover, given that a particle may traverse between the surface and the center several times prior to its ultimate detachment, we monitor the frequency of these surface-return occurrences for each particle, representing the ensemble average as $n_\mathrm{d} = \langle n_j \rangle_{j=1}^{20}$. Consequently, the effective residence time is formulated as $\tau_\mathrm{d} = [n_\mathrm{d} r_{ss}^2]/[3D_{ss}]$, resulting in a purely diffusion-based detachment frequency,

$$f_\mathrm{d} = \frac{1}{\tau_\mathrm{d}} = \frac{3D_{ss}}{n_\mathrm{d} r_{ss}^2}$$

In Fig. S12B, although the diffusion-based f_d converges towards f^- values independently obtained from the Poisson residence-time statistics, it requires the surface-return factor n_d, demonstrating that pure diffusion alone does not determine the detachment frequency. Moreover, the internal droplet diffusion coefficient D_{ss} is consistently greater than the bulk liquid diffusion coefficient D under equivalent temperature and pressure conditions, converging towards the bulk value as the cluster size r_{ss} increases. The bulk reference value is determined to be $D = 1.3$ nm^2 s^{-1} based on an independent 40 ns NVT simulation of a macroscopic liquid phase, which consists of $N = 11,264$ atoms, with a system size of $L = 8$ nm and a liquid density of 22 nm^{-3}. The marginally accelerated diffusion observed in finite droplets ($D_{ss} > D$) is likely indicative of diminished local density and reduced average coordination numbers of atoms near the interfacial region (Fig. S13D). In conclusion, hydrodynamic coupling under periodic boundary conditions yields a minor finite-size correction to the simulated bulk D [14]; however, this correction is relatively small. The incorporation of this correction does not alter the empirical observation that interfacial confinement fundamentally increases internal atomic mobility in comparison to the bulk state. The parameters

utilised for the calculation of f_{d} are presented in Table S1.

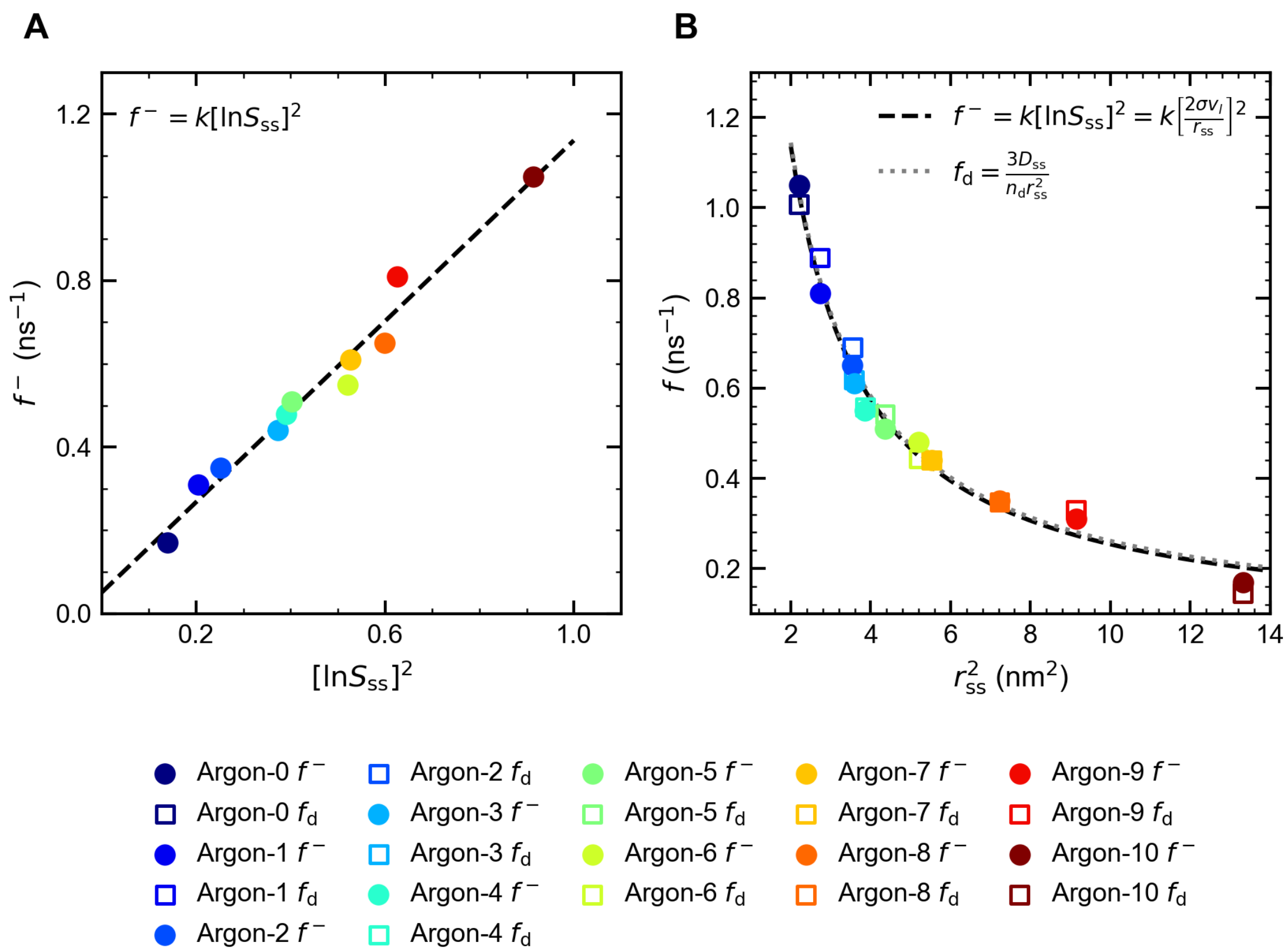

FIG. S12. (A) A linear correlation between f^-, the detachment rate in confined systems computed from the Poisson-based ECDF route, and $[\ln S(n_{ss)}]^2$. (B) f^- and f_{d}, the detachment rates computed from the diffusion-based MFPT, as a function of the square of the steady-state radius r_{ss}^2.

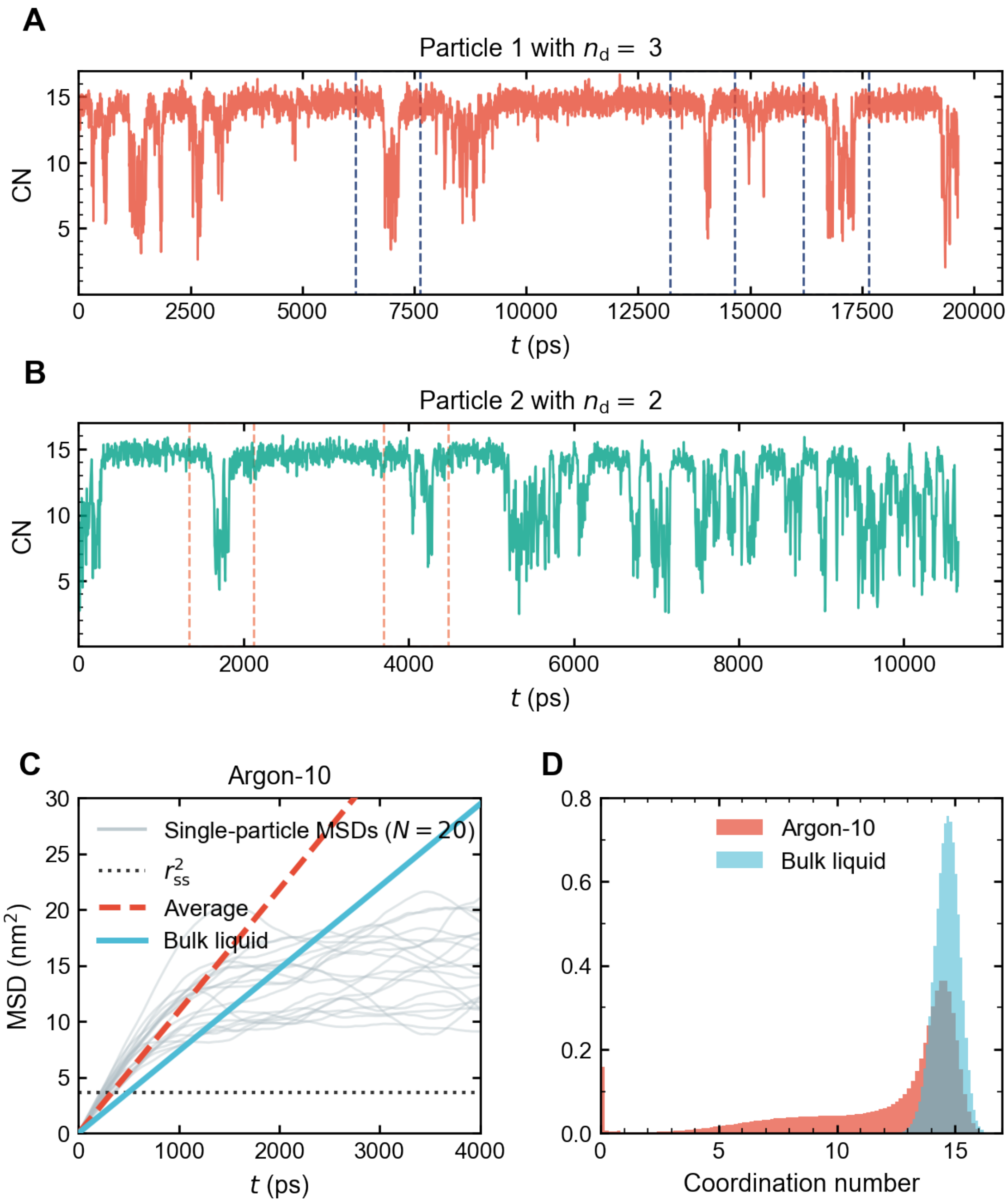

FIG. S13. (A) The evolution of the coordination number of particle 1 in the liquid droplet during its longest residence. (B) The evolution of the coordination number of particle 2 in the liquid droplet during its longest residence. (C) The mean-squared displacement of the first twenty particles in argon-10, and that of the bulk liquid in the NVT simulation. (D) The coordination number of all liquid particles in argon-10, and that of the bulk liquid in the NVT simulation.

TABLE S1. Diffusion of argon particles in the liquid cluster

i	$D_{ss,i}$ (nm^{-2} ns^{-1})	$n_{d,i}$	$r^2_{ss,i}$ (nm^2)	f_i^- (ns^{-1})	$f_{d,i}$ (ns^{-1})
0	3.4	4.6	2.2	1.05	1.01
1	3.2	4.0	2.7	0.81	0.89
2	3.1	4.3	3.5	0.65	0.62
3	2.9	3.5	3.6	0.61	0.69
4	2.9	4.0	3.9	0.55	0.56
5	3.1	3.9	4.4	0.51	0.54
6	2.9	3.8	5.2	0.48	0.44
7	2.6	3.2	5.5	0.44	0.44
8	2.5	3.0	7.2	0.35	0.35
9	2.6	2.6	9.1	0.31	0.33
10	1.8	2.8	13.3	0.17	0.15

[1] V. Ramasubramani, B. D. Dice, E. S. Harper, M. P. Spellings, J. A. Anderson, and S. C. Glotzer, freud: A software suite for high throughput analysis of particle simulation data, Computer Physics Communications **254**, 107275 (2020).

[2] K. S. Pitzer, Thermodynamics of electrolytes. i. theoretical basis and general equations, Journal of Physical Chemistry **77**, 10.1021/j100621a026 (1973).

[3] D. G. Archer, Thermodynamic properties of the nacl+h2o system. ii. thermodynamic properties of nacl(aq), nacl2h2(cr), and phase equilibria, Journal of Physical and Chemical Reference Data **21**, 10.1063/1.555915 (1992).

[4] N. E. Zimmermann, B. Vorselaars, D. Quigley, and B. Peters, Nucleation of NaCl from Aqueous Solution: Critical Sizes, Ion-Attachment Kinetics, and Rates, Journal of the American Chemical Society **137**, 13352 (2015).

[5] C. P. Lamas, J. R. Espinosa, M. M. Conde, J. Ramírez, P. M. D. Hijes, E. G. Noya, C. Vega, and E. Sanz, Homogeneous nucleation of nacl in supersaturated solutions, Physical Chemistry Chemical Physics **23**, 10.1039/d1cp02093e (2021).

[6] C. Liu, G. P. Wood, and E. E. Santiso, Modelling nucleation from solution with the string method in the osmotic ensemble, Molecular Physics **116**, 10.1080/00268976.2018.1482016 (2018).

[7] A. Llamas-Jaramillo, I. Latella, and D. Reguera, Free-energy reconstruction from stable clusters (fresc): A new method to evaluate nucleation barriers from simulation, Journal of Chemical Physics **164**, 10.1063/5.0309755 (2026).

[8] D. Reguera, R. K. Bowles, Y. Djikaev, and H. Reiss, Phase transitions in systems small enough to be clusters, Journal of Chemical Physics **118**, 10.1063/1.1524192 (2003).

[9] J. Wedekind, D. Reguera, and R. Strey, Finite-size effects in simulations of nucleation, Journal of Chemical Physics **125**, 10.1063/1.2402167 (2006).

[10] L. Li, M. Paloni, A. R. Finney, A. Barducci, and M. Salvalaglio, Nucleation of Biomolecular Condensates from Finite-Sized Simulations, Journal of Physical Chemistry Letters **14**, 1748 (2023).

[11] R. C. Tolman, The effect of droplet size on surface tension, The Journal of Chemical Physics **17**, 10.1063/1.1747247 (1949).

[12] M. Camarillo, I. Sanchez-Burgos, C. P Lamas, P. Montero de Hijes, J. R Espinosa, and E. Sanz, Condensation vs cavitation in water: A simulation study, The Journal of Chemical Physics **163** (2025).

[13] A. E. V. Giessen and E. M. Blokhuis, Direct determination of the tolman length from the bulk pressures of liquid drops via molecular dynamics simulations, Journal of Chemical Physics **131**, 10.1063/1.3253685 (2009).

[14] I. C. Yeh and G. Hummer, System-size dependence of diffusion coefficients and viscosities from molecular dynamics simulations with periodic boundary conditions, Journal of Physical Chemistry B **108**, 10.1021/jp0477147 (2004).
